# Unveiling the pharmacological mechanisms of *Spirulina platensis* in rheumatoid arthritis rats through the integration of serum metabolomics, pathways analysis, and experimental validation

**DOI:** 10.1007/s00210-025-04191-y

**Published:** 2025-05-07

**Authors:** Dina S. Ghallab, Eman Shawky, Asmaa A. Khalifa, Samar S. Elblehi, Mohamed M. Mohyeldin, Reham S. Ibrahim

**Affiliations:** 1 Department of Pharmacognosy, Faculty of Pharmacy, Alexandria University, Alexandria, Egypt; 2 Department of Pharmacology and Therapeutics, Faculty of Pharmacy, Pharos University in Alexandria, Alexandria, Egypt; 3 Department of Pathology, Faculty of Veterinary Medicine, Alexandria University, Alexandria, Egypt

**Keywords:** Rheumatoid arthritis, *Spirulina platensis*, Serum metabolomics, Anti-inflammatory, Mechanism of action

## Abstract

Rheumatoid arthritis (RA) is an autoimmune disease primarily manifested by insistent proliferative synovitis, joint degradation, and bone erosions with no targeted therapy yet. *Spirulina platensis* serves as a treasure house of bioactive compounds with potential significance against different inflammatory ailments. Inspired by the potentiating biological attributes of *S. platensis*, the current investigation is concerned with dissecting the mechanistic basis of *S. platensis* against rheumatoid arthritis (RA) through a series of biochemical and histopathological assessments integrated with a serum metabolomics strategy to explore more efficacious and safe alternative therapies to rectify RA. Firstly, a rat model of RA was established using complete Freund’s adjuvant (CFA), and RA-related biochemical and histopathological scores were determined as monitoring indexes for control efficiency of *S. platensis* against RA. Serum metabolomics was adopted to profile the potential biomarkers and their corresponding metabolic pathways modulated by *Spirulina* through UPLC-MS/MS analysis integrated with chemometrics and MetaboAnalyst 5.0 pathway analysis. The results demonstrated that *Spirulina* exerted significantly modulatory effects in the CFA model by reducing systemic manifestations of oxidative stress, inflammation, and impaired liver and kidney functions typically exemplified by catalase (CAT), superoxide dismutase (SOD), reduced glutathione (GSH), rheumatoid factor (RF), monocyte chemotactic protein 1 (MCP-1), tumor necrosis factor alpha (TNF-α), and interleukin-6 (IL-6), as well as alanine aminotransferase (ALT), aspartate aminotransferase (AST), creatinine, and urea. Histopathological investigations have revealed that *Spirulina* intervention causes moderately lower inflammatory cells infiltrations, synovial hyperplasia, and cartilage destruction. Regarding serum metabolomics, *Spirulina* could remarkably reverse disordered RA-associated metabolites, namely glutamic acid, arachidonic acid, 5-hydroxyeicosatetraenoic acid, (20:4/18:0) phosphatidylcholine, and citric acid, to a normal-like state through modulating arachidonic acid metabolism, alanine, aspartate and glutamate metabolism, and citrate cycle pathways putatively implicated in inflammation and joint damage. Our findings provide compelling evidence that* S. platensis* possesses a broad spectrum of mechanisms to restore the disrupted homeostasis in RA by multi-targeted, synergistic actions.

**Graphical abstract:**

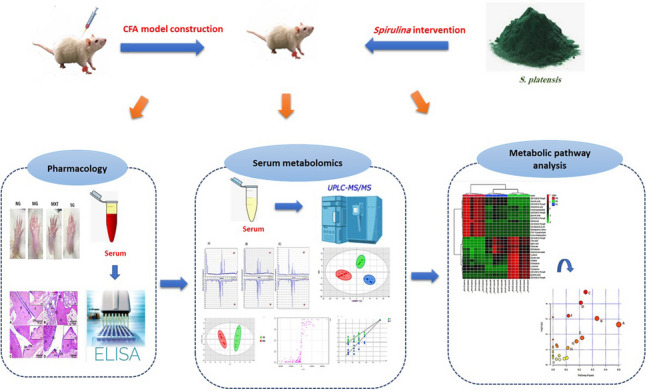

**Supplementary Information:**

The online version contains supplementary material available at 10.1007/s00210-025-04191-y.

## Introduction

Rheumatoid arthritis (RA) is an autoimmune disease primarily manifested by insistent proliferative synovitis, joint degradation, and bone erosions (Giannini et al. 2020). Non-steroidal anti-inflammatory drugs (NSAIDs), glucocorticoids, and disease-modifying anti-rheumatic drugs (DMARDs) offer a renowned group of current medications largely prescribed in the symptomatic treatment of RA (Bartikoski et al. 2022). However, concerns regarding their security and tolerability have grown with a more careful evaluation of pharmacovigilance ponders in recent years (Bartikoski et al. 2022). Furthermore, the appearance of adverse consequences such as gastrointestinal bleeding and cardiovascular toxic effects for NSAIDs, hepatotoxicity, an interstitial pulmonary disease for DMARDs, Cushing syndrome, and diabetes for glucocorticoids reduces the patient adherence to these medications (Bartikoski et al. 2022). Thus, there is a strong need to investigate safer and more effective alternative treatments to deal with this crippling illness.

Undoubtedly, oceans comprising approximately 70% of the planet’s surface harbor an unlimited variety of organisms that offer a broad spectrum of unique molecular scaffolds with promising biological activities (Greer et al. 2020). Among marine organisms, microalgae represent pioneering photosynthetic organisms with great morphological, genetic, and biochemical diversity.

*Spirulina platensis* represents one of the most promising blue-green microalgae matrices packed with valuable compounds with potential nutraceutical significance (Ghallab et al. 2022b). From a chemical point of view, *S. platensis* serves as a treasure house of bioactive compounds, primarily fatty acids, carotenoids, phenolic compounds, vitamins, and many others with a broad range of biological attributes, such as anti-inflammatory, anti-cancer, antioxidant, antidiabetic, antimicrobial, and wound-healing properties (Wang et al. 2017).

Intriguingly, *Spirulina is* frequently consumed as a staple food in many Asian nations and has ethnopharmacological value in traditional Chinese medicine for managing a wide range of inflammatory conditions (Chu 2012; Liu et al. 2022). In a more targeted approach, some earlier reports demonstrated the pronounced anti-arthritic action of *S. platensis* in animal models (Kumar et al. 2009; Ali et al. 2015). For the sake of clarity, the oral administration of 400 mg/kg of *S. platensis* to CIA rats resulted in a significant increase in serum albumin and a remarkable decrease in serum cholesterol, lipid peroxidation, paw thickness, and joint histopathology, inferring the promising protective efficacy of *S. platensis* against RA (Kumar et al. 2009). Another study has demonstrated the noteworthy anti-angiogenic and immunomodulatory potential of *S. platensis* in a complete Freund’s adjuvant-induced arthritis (CFA) model (Ali et al. 2015) via lowering serum levels of COX- 2, TNF-α, IL- 6, and VEGF compared to the RA group (Maryati et al. 2020).

Metabolomics can provide a complete overview of metabolic status and the dynamic alterations of metabolites throughout the entire biosystem triggered by disease interference or environmental undulations (Idle and Gonzalez 2007). Presently, the use of such a systematic technique to identify the hallmark metabolites in cells, organs, or excretion can identify the pathway linked to the development and progression of human diseases and prospectively assess the effectiveness of herbal remedies (Weckwerth 2003). Serum metabolomics has been thrivingly employed for discovering the systematic biological characteristics remarkably correlated with diseases, for precisely understanding the essence of diseases, and for reflecting the overall role of drugs in treating complex diseases (Zhang et al. 2012).

Despite these studies regarding the significance of *Spirulina* as a complementary medicine against RA, the efficacy mechanisms of *Spirulina* on RA at the metabolic level persist in lagging and call for more research. So far, several considerations must be addressed in the research community to elaborate on how *Spirulina* acts against RA, hoping to move forward to the investigation of new and more effective RA treatment strategies. Furthermore, exploring the metabolic pathways associated with RA might be a targeted therapeutic strategy to shut down unwarranted immune reactions and manage RA.

To address these concerns, the research strategy was approached to systematically throw light on the detailed therapeutic mechanism of *S. platensis* against RA from a serum metabolomics perspective. Correspondingly, a rat model of RA was created utilizing CFA for assessing *S. platensis* control efficacy on RA. Efficacy evaluation was then performed through a series of biochemical and histopathological scores. Furthermore, a UPLC-MS-based metabolomics strategy coupled with chemometric analyses was implemented to monitor the variations in RA-related metabolites and their pathways, deciphering the multi-targeted mechanisms of *Spirulina* against RA. As far as we know, the mechanistic basis whereby *S. platensis* can alleviate RA has yet to be fully clarified, especially from a metabolomics perspective. The data gathered from this investigation will provide a strong basis for a wider and more rational clinical use of *S. platensis* in the supportive management of RA.

## Materials and methods

### Chemicals and reagents

CFA (10 mg/mL) methotrexate, used as a positive control, was acquired from Sigma-Aldrich, Germany. Sodium carboxymethyl cellulose (Na-CMC) was gained from Arabic Laboratory Equipment Co., Cairo, Egypt. Citric acid, glutamic acid, and arachidonic acid as external standards were purchased from Sigma-Aldrich (St. Louis, MO, USA).

### Preparation of Spirulina platensis extract

Early in the summer of 2022, a well-known cyanobacterium, *Spirulina platensis*, was collected fresh from the coastal region of Abu-Qir Alexandria, North Egypt, and taxonomically confirmed using Algae Base (http://www.algaebase.org). Subsequently, the microalgal sample was cleansed with double-distilled water to confiscate unwanted adhering contaminants like sand, salt deposits, and epiphytes and dried at room temperature in the shade for 10 days.

Following our previous optimized protocol (Ghallab et al. 2022b), the dried specimen was finely powdered using an electric blender, sifted (50-mesh), accurately weighed, and subjected to extraction (twice) with 70% ethanol in an ultrasonic bath apparatus (3L Alpha Plus, Japan) at 40 °C for 2 h. Then, all filtrates were concentrated to dryness under a vacuum using a rotary evaporator at 40–50 °C, subjected to freeze-drying, and preserved at 4 °C for future use.

For pharmacological investigations, distilled water with 1% w/v Na-CMC was used to suspend the spirulina extract. The doses utilized are stated as mg of dry extract per kg body weight (Lin et al. 2014; Ghallab et al. 2024a).

### Animals

Thirty-two 150–200 g Sprague–Dawley rats were acquired from the animal house of Pharos University’s Faculty of Pharmacy in Alexandria. Details regarding the age and weight of each rat are provided in Table S1.

Rats were housed on wood shavings in stainless-steel cages for animals, with eight rats per cage. Rats were kept at 20–22 °C in a lighting/dark time of 12:12 h cycle under optimal sanitary conditions and allowed free access to water and food all day and throughout the study. All interventions were carried out with a minimal amount of distress throughout the experiment. The study was conducted in compliance with the National Institutes of Health’s “National Research Council’s Guide for the Care and Use of Laboratory Animals” (Couto and Cates 2019).

### Establishment of rheumatoid arthritis rats model and treatment

All procedures in this study were approved by the Institutional Animal Care and Use Committee (IACUC), Alexandria University (Approval No. 0620228112123).

A detailed description for a small-scale preliminary study initially directed to convincingly select the optimal therapeutic dose of *S. platensis* extract being used in our recent investigation was clarified in the supplementary material.

The induction of RA was made by injection of CFA (0.1 mL, subcutaneous injection) into the plantar region of the right hind paw of the rats. Two booster doses of CFA (0.1 mL, intradermal injection) were given into the root of the tail; the first booster dose was given 1 h following the intra-plantar injection, and the second one was given on the next day. Edema appeared within 24 h of CFA injection. The arthritis induction day was designed as day 0, and significant joint inflammation was observed by day 14 (Abdel-Maged et al. 2019). In the current study, 4 weight-matched groups (6 rats/group) were utilized and divided as follows: group I (normal): containing the negative control rats. Group II (RA model): including the untreated arthritic rats. Group III (MTX): containing the arthritic rats treated with the standard methotrexate (MTX) (0.75 mg/kg/week, intraperitoneal) (Paulos et al. 2006). Group IV (SP): including the arthritic rats treated with *S. platensis* extract (400 mg/kg/day, oral, dissolved in 5 mL of 1% CMC). The treatments in groups III and IV were started on day 14 and continued for 28 days until day 42 (Tekeoğlu et al. 2020).

### Measurement of hind paw edema

The edema that exists in the right hind paw was used to evaluate the progression of arthritis in rats. To confirm the induction of the CFA and the outcome of treatments, the tibiotarsal joint width was measured using a caliper device (Dasqua Co., Italy) on days 0, 7, 14, 21, 28, and 42 in all rats in the study. Before killing, representative pictures were taken to show the impact of treatments used in the current study.

### Measurement of serum parameters

A colorimetric assay was used for the detection of urea, creatinine, superoxide dismutase (SOD) activity, malondialdehyde (MDA) concentration, catalase activity, and reduced glutathione (GSH) using the commercially available kits (Biovision Incorp., USA, catalog No. K376, K625, K335, K739, K773, and K464, respectively). In addition, the ELISA kits were used to detect the following serum parameters: alanine aminotransferase (ALT) and aspartate aminotransferase (AST) (Biomatik Co., USA, catalog No. EKE62019 and EKU02211, respectively), rheumatoid factor (RF) (Wuhan Fine Biotech Co., China, catalog No. ER1932), monocyte chemotactic protein 1 (MCP- 1) (Cloud-Clone Corp., USA, Catalog No. SEA087Ra), tumor necrosis factor alpha (TNF-α), (Biolegend Co., USA, catalog No. 438205), and interleukin- 6 (IL- 6) (Cloud-Clone Corp., USA, catalog No. SEA079Ra).

### Histopathological examination and semi-quantitative lesion scoring

Right hind paws were removed from eight animals per group and fixed overnight in 10% buffered neutral formalin (10%, pH 7.4) for 2 days, followed by decalcification in 10% formic acid for 5 to 7 days. Decalcified tissues were embedded in paraffin and sliced in a mid-sagittal plane into 4 µm thickness, placed on slides, xylene deparaffinated, and ethanol rehydrated. Sections were stained with hematoxylin and eosin (H&E) for a general assessment of the degree of synovitis, hyperplasia of the synovial lining cells and pannus formation, inflammatory cell infiltrations, and bone and/or cartilage destruction. Tissue slides were inspected in a blinded manner to prevent bias using a light microscope (Leica, DM500) at a magnification power of × 100 and × 400 and snapped using a digital camera (EC3, Leica, Germany). A semi-quantitative lesion score was adapted to grade the histopathological changes in the CFA joints based on the intensity and extent of histological changes to the ankle joints using a score between 0 and 3 scale (El-Ghazaly et al. 2020). The severity of microscopic arthritic changes, including synovial hyperplasia, synovial vascularity, infiltration of inflammatory cells, pannus formation, cartilage erosion, and bone erosion, was evaluated in H&E-stained slides using the following grades: 0 = no abnormality detected, 1 = mild, 2 = moderate, and 3 = severe.

### Serum metabolomics

#### Preparation of metabolic serum samples

After the CFA test, rats were humanely killed under anesthesia with 1% thiopental sodium anesthesia (30 mg/kg, intraperitoneal injection). Blood was collected through the abdominal aorta and the serum was subsequently separated by centrifugation (3500 × *g*, 15 min, 4 °C), and stored at − 80 °C until use. Serum samples were prepared for LC/MS analysis by protein precipitation with methanol. Briefly, 1 mL of thawed serum and 3 mL of chromatography-grade methanol were mixed evenly, vortexed for 60 s, and centrifuged for 10 min at 4 °C and 12,000 rpm to remove proteins and particles. The supernatant was then dried using nitrogen gas after being filtered through a microporous membrane (0.22 μm). The residue was redissolved in 100 μL of methanol, and 5 μL was loaded onto the chromatographic column.

#### Acquisition of LC–MS data

Metabolomics analysis of serum samples was illustrated following our previous protocols (Ghallab et al. 2022a, 2024b, 2024c).

In brief, metabolomics analysis of serum samples was conducted on a UPLC XEVO TQD triple quadruple instrument (Waters Corporation, Milford, MA01757, USA). Serum chromatographic separation was conducted using a Waters Acquity UPLC BEH C18 column (50 × 2.1 mm ID × 1.7 μm particle size) with temperature maintained at 35 °C. The binary mobile phase was comprised of acidified ultrapure water (0.1% formic acid) (phase A) and acidified methanol (0.1% formic acid) (phase B) and was gradient eluted at a flow rate of 0.2 mL min^−1^. Of note, the gradient elution program and operational parameters for the ESI interface were fully described in the supplementary material.

Importantly, a 10-µL aliquot of each plasma sample was mixed to acquire a quality control (QC) sample for method validation. The QC sample was injected five times at 2, 4, 6, 8, 12, and 24 h to monitor the performance of the analytical method and instrument stability. The precision, repeatability, and stability were evaluated by calculating the relative standard deviation (RSD) of the peak area and retention time for five ions selected in both negative and positive ion modes.

MZmine 2.0 (http://mzmine.sourceforge.net/), qualitative analysis software, was used for data acquisition and processing. Subsequently, a data matrix containing the retention time, mass-to-charge ratio (*m/z*), and peak intensity was obtained.

### Multivariate data analysis

For multivariate analysis, the obtained results were then imported into SIMCA-P software (version 14.1; Umetrics, Umea, Sweden). Principal component analysis (PCA) was initially applied to get a general overview of the metabolic data and visualize any outliers, trends, or general intrinsic patterns. Then, to create a variable identification model and maximally examine the discrepancies between the comparable groups, orthogonal projection to latent structure-discriminant analysis (OPLS-DA) was constructed.

The contributions of the metabolites to the change rates of *Y* variables in OPLS-DA were described using the variable importance of projection (VIP) values. The metabolites with VIP > 1 showed a significant commitment to group classifications.

These variables were also subjected to univariate analysis using the *t*-test and fold-change, where variables with significant coefficient *p* values < 0.05 and fold-changes (FC) > 2.0 (or < 0.5) were picked out. As a result, metabolites that satisfied the criteria VIP > 1, FC > 2.0 (or < 0.5), and *p* values < 0.05 were ultimately chosen as “differential metabolites” or “potential markers” for further appraisal.

Based on the respective *R*^2^ and *Q*^2^, the models’ predictive reliability was assessed. Additionally, the overfitting of OPLS-DA models was evaluated using permutation tests (*n* = 20), which confirmed the precision of the putative diagnostic biomarkers.

The following online mass spectrometry databases, HMDB (http://www.hmdb.org/) and METLIN (http://metlin.scripps.edu/) were utilized to profile possible biomarkers. Reference standards, diagnostics tandem mass profiles, and related literature were consulted as well.

### Metabolic pathways analysis

MetaboAnalyst 5.0 (https://www.metaboanalyst.ca/) was used to unveil the metabolic pathways impacted by RA and regulated by *Spirulina* based on significantly changed metabolites (VIP > 1.0, FC > 2.0 (or < 0.5), and *p* < 0.05).

The pathway library of the Small Molecule Pathway Database (SMPDB) was selected, and the metabolic pathway enrichment of potential metabolites was performed using the KEGG database (https://www.kegg.jp/). Admittedly, the pathway impact factor was evaluated by the relative relevance of the metabolites where the pathway was deemed substantially connected if its impact value was greater than 0.1 and its *p* value was less than 0.05.

### Statistical analysis

Data were presented as the mean ± SD of eight rats per group. GraphPad Prism v8.0 (La Jolla, CA, USA) was used to evaluate the values. Tukey’s test for post hoc analysis and multiple comparisons was employed after a one-way ANOVA to compare the four groups; a *p* value of less than 0.05 was considered statistically significant.

## Results and discussion

### Key ingredients in S. platensis extract

Table S2 summarizes the entire list of metabolites derived from the *S. platensis* extract analyzed. These metabolites were broadly classified into various phytochemical classes, such as amino acids, phenolic acids, flavonoids, fatty acids and their derivatives, and carotenoids.

Additionally, Fig. S1 shows the base peak chromatograms (BPCs) of the examined *S. platensis* sample in positive and negative ionization modes.

*S. platensis* serves as a prime reserve of different biologically active compounds with undeniable bio-functionalities.

### The small-scale preliminary study for optimal selection of S. platensis extract dose

As alluded to above, to rationally select the optimal therapeutic dosage of *S. platensis* extract for our current study, a small-scale preliminary study evaluating the anti-arthritic actions of three dosages of the extract was conducted. Table S3 and Fig. S2 present the findings of this small-scale study.

In short, in alignment with our preliminary findings monitoring arthritis scores along with some supportive studies (Kumar et al. 2009; Ali et al. 2015), it was observed that *S. platensis* dose (400 mg/kg/day) effectively restored the perturbations of CFA rats close to the normal state as well as rectified the joint histopathology of arthritic rats.

Notably, consistent with previous toxicological reports, both short- and long-term ingestions of *S. platensis*—up to 10 g/kg body weight—have been shown to cause no toxicity or negative reactions in experimental animals (Hutadilok-Towatana et al. 2008). Additionally, a sub-chronic toxicity study was performed on groups of ten mice of each sex that were given *S. platensis* for 13 weeks at concentrations of 0 (control), 10, 20, or 30% (w/w). The findings demonstrated that *S. platensis* did not have any negative effects on mice when fed at high levels (Hutadilok-Towatana et al. 2010). Importantly, *Spirulina* is now included in the list of drugs classified as Generally Recognized as Safe (GRAS) by the US Food and Drug Administration (ElFar et al. 2022).

### Effects of S. platensis on hind paw swelling

**Fig. 1:**
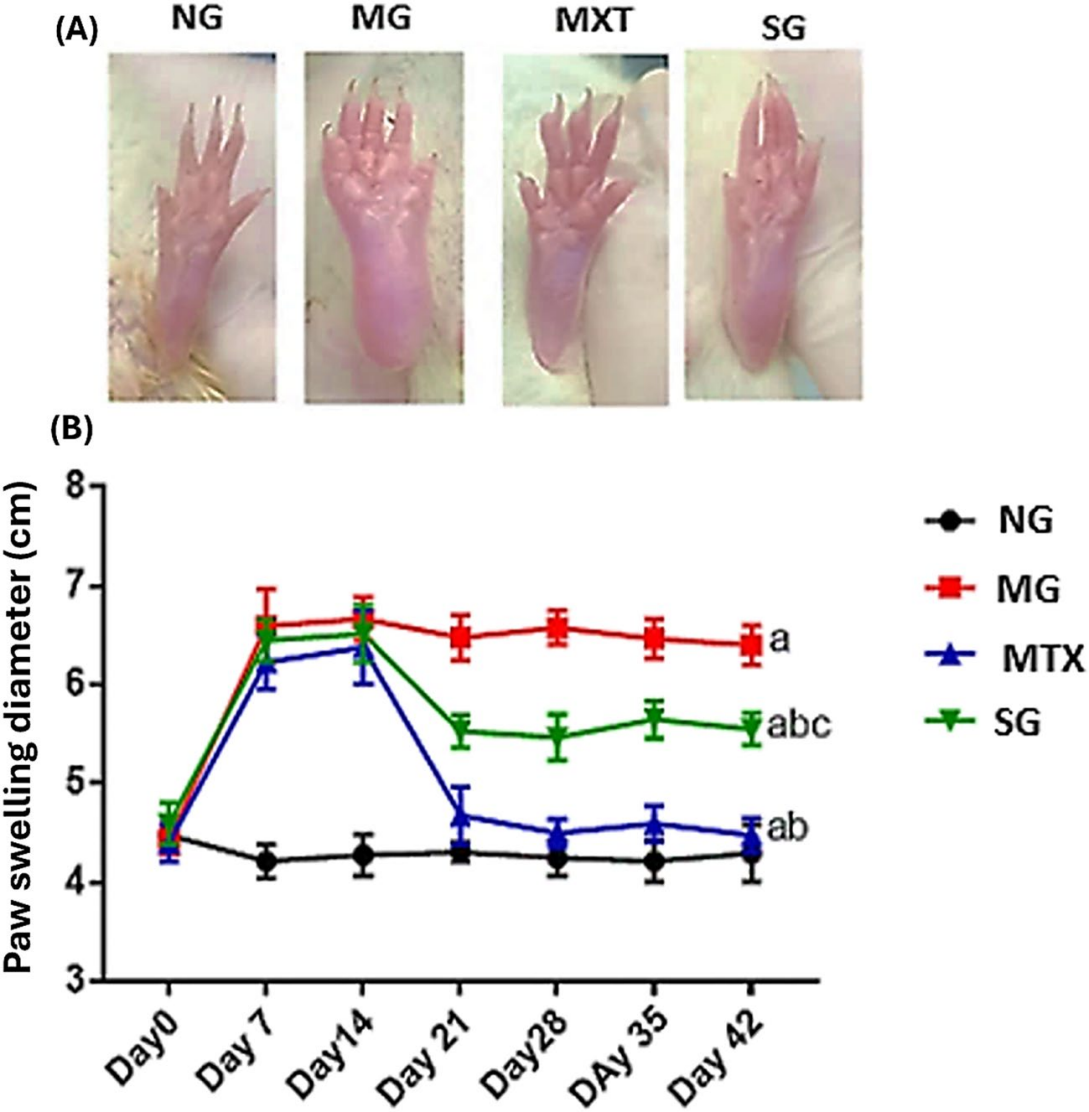
**A** Representative photos showing the appearance of the right hind paw swelling in rats from the normal, RA model, MTX, and *Spirulina* groups. **B** Line curves depict changes in right hind paw diameter at 0, 7, 14, 21, 28, 35, and 42 days after CFA injection in the respective groups. NG, normal group; MG, RA model group; MTX, methotrexate-treated rats; SG, *Spirulina* extract-treated group. MTX and SP were administrated for 28 days following the induction of arthritis. Values expressed as mean ± SD (*n* = 6). Lowercase letters indicate significant differences compared with normal rats (a), RA rats (b), and MTX-treated rats (c) at *p* ≤ 0.05, determined by one-way ANOVA followed by Tukey’s post hoc test

Figure 1A clearly shows the impact of treatments on the rats that received the MG injection. In arthritic rats, a progressive significant increase in hind paw swelling on day 14 as compared to day 0 (6.67 ± 0.22 vs. 4.44 ± 0.15), respectively (Fig. 1B). In the untreated arthritic rats, the hind paw swelling increased significantly by 49% on day 42 as compared to the control group. Conversely, a significant decline in the hind paw swelling in arthritic rats was recorded after the administration of MTX or SP from days 14 to 42, by 30 and 13%, respectively, compared with the RA group (Fig. 1B), indicating that *S. platensis* extract could relieve the pain and inflammation in arthritic rats.

### Effects of S. platensis on the serum biochemical indices

#### Effect of Spirulina on the serum markers of liver and kidney functions of RA rats

Kidney and liver dysfunction is intimately linked with RA disease activity and bone erosion progression (Tang et al. 2021). Accordingly, ALT, AST, creatinine, and urea are mainly used as monitoring indices of liver and kidney impairment (Tang et al. 2021). As demonstrated in Table 1, the untreated arthritic rats demonstrated a significant elevation in serum liver enzymes ALT and AST compared with the control rats. Comparing the MTX group to the RA group, the serum levels of ALT and AST increased significantly by 50 and 68%, respectively. On the contrary, the serum samples obtained from the rats treated with *S. platensis* extract revealed no significant alterations in the liver function markers as compared with the normal group. Similar effects were noticed during the evaluation of the serum markers of kidney functions. The untreated arthritic rats showed a significant elevation of serum urea and creatinine when compared to the control rats. In addition, the administration of MTX to the arthritic rats caused a more significant rise in the serum urea and creatinine by 39 and 150%, respectively, as compared to the untreated arthritic rats. However, the serum obtained from *S. platensis* extract-treated rats showed no significant elevation when compared to serum obtained from the control group (Table 1).

**Table 1:** Effect of *S. platensis* extract on serum markers of liver and kidney function in RA rats

Serum	Normal (NG)	RA model (MG)	MTX	SG
ALT (ng/mL)	0.60 ± 0.08	1a ± 0.15	1.5ab ± 0.16	0.74bc ± 0.06
AST (ng/mL)	1.4 ± 0.04	2.2a ± 0.14	3.7ab ± 0.13	1.6bc ± 0.1
Urea (µmol/mL)	21 ± 2.4	49.2a ± 2.5	68.5ab ± 4.7	35.2bc ± 2.7
Creatinine (µmol/mL)	0.65 ± 0.09	1a ± 0.09	2.5ab ± 0.15	0.74bc ± 0.11

*NG*, normal group; *MG*, RA model group; *MTX*, methotrexate-treated rats; *SG*, *Spirulina* extract-treated group; *ALT*, alanine aminotransferase; *AST*, aspartate aminotransferase. Values are means ± SD (*n* = 6), compared with control (a), RA (b), and MTX (c), using one-way ANOVA followed by Tukey’s post hoc test at *p* ≤ 0.05

Undoubtedly, MTX is one of the most efficient chemotherapeutic drugs that has been used traditionally to treat autoimmune and malignant disorders in millions of patients (Khafaga and El-Sayed 2018). It is regarded as the most often prescribed DMARD for RA patients worldwide (Khafaga and El-Sayed 2018). Nevertheless, hepatotoxicity and nephrotoxicity are the main adverse effects associated with MTX intervention (Khafaga and El-Sayed 2018). Thus, in the current investigation, treatment with MTX demonstrated a sharp rise in serum liver enzymes ALT and AST as well as urea and creatinine compared with the control rats. Instead, *Spirulina* supplementation maintained normal liver and kidney functions and greatly guarded against alterations in these parameters.

Even though the precise mechanism underlying MTX’s adverse effects is not well understood, several theories have been proposed that oxidative stress and inflammation may play a role in such pathogenesis. Worthy to mention, *Spirulina* has exceptional anti-inflammatory, antioxidant, and immune-stimulatory qualities that may guard against MTX-induced nephrotoxicity and hepatotoxicity. Accordingly, *Spirulina* could be considered a safer, more effective option in the arsenal of RA control tools.

#### Effect of Spirulina on serum oxidative stress and inflammatory indices of RA rats

Inconsistency of the oxidative system in RA intensifies oxidative stress, concluding with the destruction of cartilage and bone. Thus, endogenous antioxidant enzymes, primarily CAT, SOD, and GSH, are enhanced to offer a first line of defense against oxidative damage in RA. As illustrated in Table 2, the use of CFA injection for the induction of arthritis in the RA group resulted in a significant elevation of the oxidative stress parameters in the serum of arthritic rats when compared to the normal group. All serum oxidative stress indices were improved in MTX- or SP-treated rats as compared with non-treated rats in the RA group. The MDA, a lipid peroxidation indicator, decreased significantly in MTX- and SP-treated groups by 65 and 42%, respectively, as compared to untreated arthritic rats. In contrast, the serum levels of the antioxidant enzymes SOD, CAT, and GSH rose dramatically in rats treated with MTX by 119, 253, and 150%, respectively, and in rats treated with SP by 61, 121, and 95%, respectively, after being greatly consumed in CFA-challenged rats, suggesting that *Spirulina* can effectively maintain the oxidative flux. Furthermore, the serum rheumatoid factor (RF) was significantly increased in non-treated RA rats. Compared with the RA group, the RF level decreased significantly by 60% in the MTX-treated rats (Table 2). Likewise, the level of RF in SP-treated rats was low compared with the untreated arthritic rats, suggesting that *S. platensis* had a profound improvement effect on oxidative stress indicators and pro-inflammatory state closely associated with disease activity in RA. Renowned for its role in RA pathogenesis, MCP- 1 is a master regulator of the recruitment of monocytes and macrophages (Deshmane et al. 2009). As such, serum MCP- 1 was markedly accumulated in CFA-challenged rats; however, it decreased significantly in the SP-treated rats by 46% as compared to the normal group. Undoubtedly, IL- 6 and TNF-α are proinflammatory cytokines that tightly contribute to joint inflammation in RA (Mori et al. 2011). In the same vein, the inflammatory cytokines TNF-α and IL- 6, excessively released in the RA group, witnessed a marked drop in MTX-treated rats by 71 and 62%, respectively. Likewise, these parameters were dramatically declined in the SP-treated group by 48 and 36%, respectively, compared with the arthritic group (Table 2**)**.

**Table 2:** Effect of *S. platensis* extract on serum oxidative stress and inflammatory indices in RA rats

Serum	Normal (NG)	RA model (MG)	MTX	SG
MDA (nmol/mL)	0.30 ± 0.02	1.9a ± 0.03	0.66ab ± 0.04	1.1abc ± 0.04
SOD (U/mL)	2.5 ± 0.19	0.93a ± 0.12	2.04ab ± 0.04	1.5abc ± 0.05
CAT (mU/mL)	2.7 ± 0.11	0.68a ± 0.04	2.4ab ± 0.06	1.5abc ± 0.04
GSH (nmol/mL)	1.5 ± 0.02	0.56a ± 0.09	1.4ab ± 0.06	0.89abc ± 0.05
RF (IU/mL)	5 ± 0.73	15.8a ± 0.62	6.3ab ± 0.38	9.4abc ± 0.93
MCP- 1 (pg/mL)	28.7 ± 6	163.5a ± 4.2	41.3ab ± 3.5	87.6abc ± 7
TNF-α (pg/mL)	27.3 ± 3.4	159.5a ± 3.1	60ab ± 2.3	103.3abc ± 7.8
IL- 6 (pg/mL)	27.6 ± 2.4	112.3a ± 10	38.5b ± 1.4	57.5abc ± 4.3

*NG*, normal group; *MG*, RA model group; *MTX*, methotrexate-treated rats; *SG*, *Spirulina* extract-treated group; *MDA*, malondialdehyde; *SOD*, superoxide dismutase; *CAT*, catalase; *GSH*, reduced glutathione; *RF*, rheumatoid factor; *MCP- 1*, monocyte chemoattractant protein- 1; *TNF-α*, tumor necrosis factor-alpha; *IL- 6*, interleukin 6. Values are means ± SD (*n* = 6), compared with control (a), RA (b), and MTX (c), analyzed using one-way ANOVA followed by Tukey’s post hoc test at *p* ≤ 0.05

#### Effect of S. platensis on histo-architecture of RA rats

**Fig. 2:**
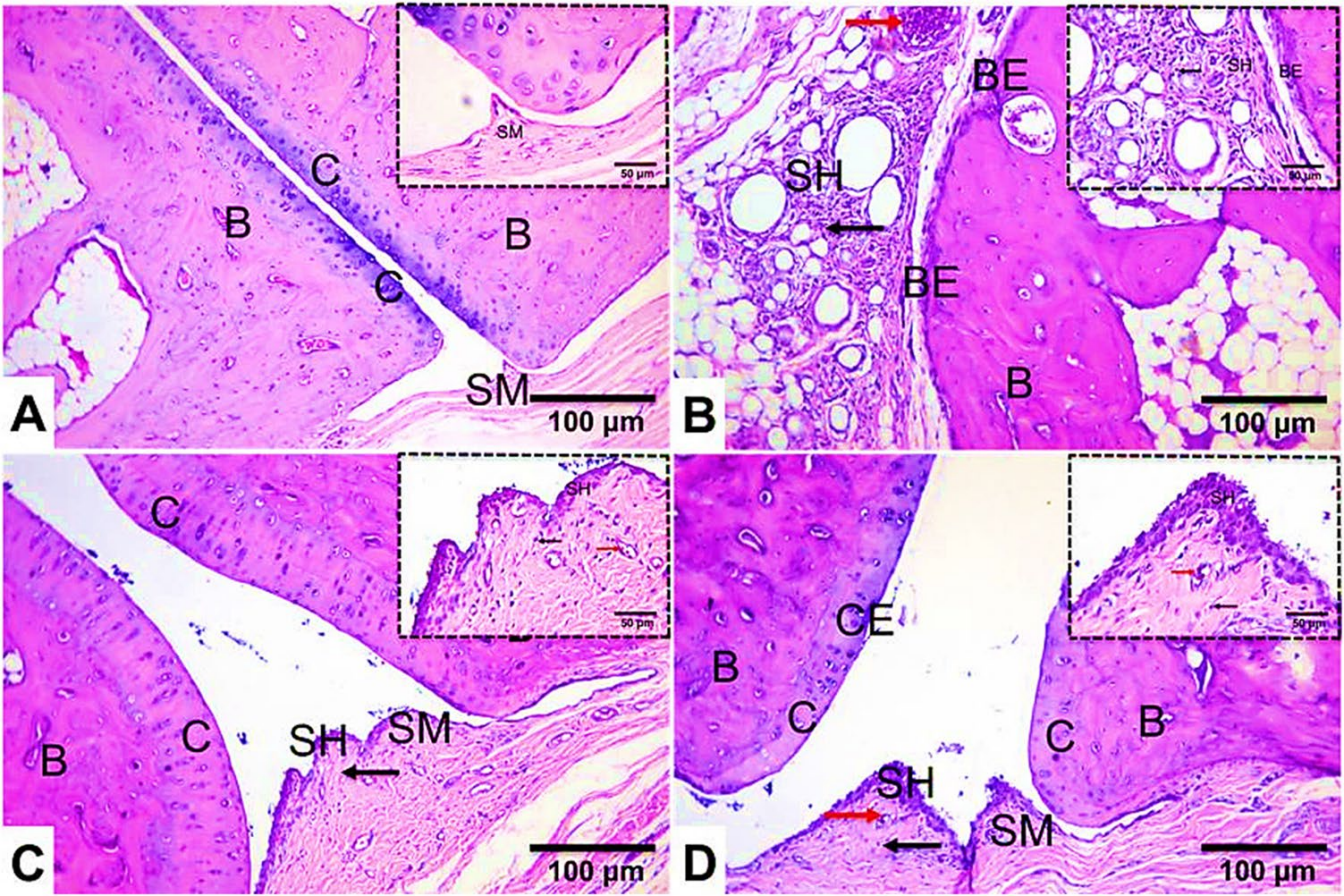
Representative photomicrographs of ankle joints from different experimental groups (*n* = 6), stained with H&E at low (× 100) and high (× 400) magnifications. **A** Joint section from a normal rat. **B** Joint section from an arthritic rat showing intense and diffuse inflammatory cells infiltrations, cartilage and bone erosion. **C** Joint section from an MTX-treated arthritic rat showing marked amelioration of pathological changes in the cartilage, with preserved bone, minor inflammatory cells infiltrations, and absence of pannus or fibrosis. **D** Joint section from an *S. platensis* extract-treated arthritic rat showing moderate amelioration of pathological changes. Spongy bone (B), articular cartilage (C), synovial membrane (SM), synovial hyperplasia (SH), synovial vascularity (red arrow), inflammatory cells infiltrations (black arrow), cartilage erosion (CE), and bone erosion (BE)

Examination of the ankle joint of normal control rats showed intact morphology. Clear joint space, normal synovium, and a thin layer of quiescent cells of the synovial lining were evident. No inflammation or influx of inflammatory cells was observed. Smooth, organized, and continuous cartilage surface lining of the joint surfaces without any bone or cartilage destruction was also noticed (Fig. 2A). Conversely, sections of untreated RA rats exhibited severe synovitis (Fig. 2B), characterized by diffuse inflammatory cells infiltrations and fibroblasts into the joint cavity, moderate enlargement of the synovial membrane, increased synovial vascularity, and synovial cell proliferation with pannus formation. Also, cartilage destruction and bone erosion were prominent findings.

**Fig. 3:**
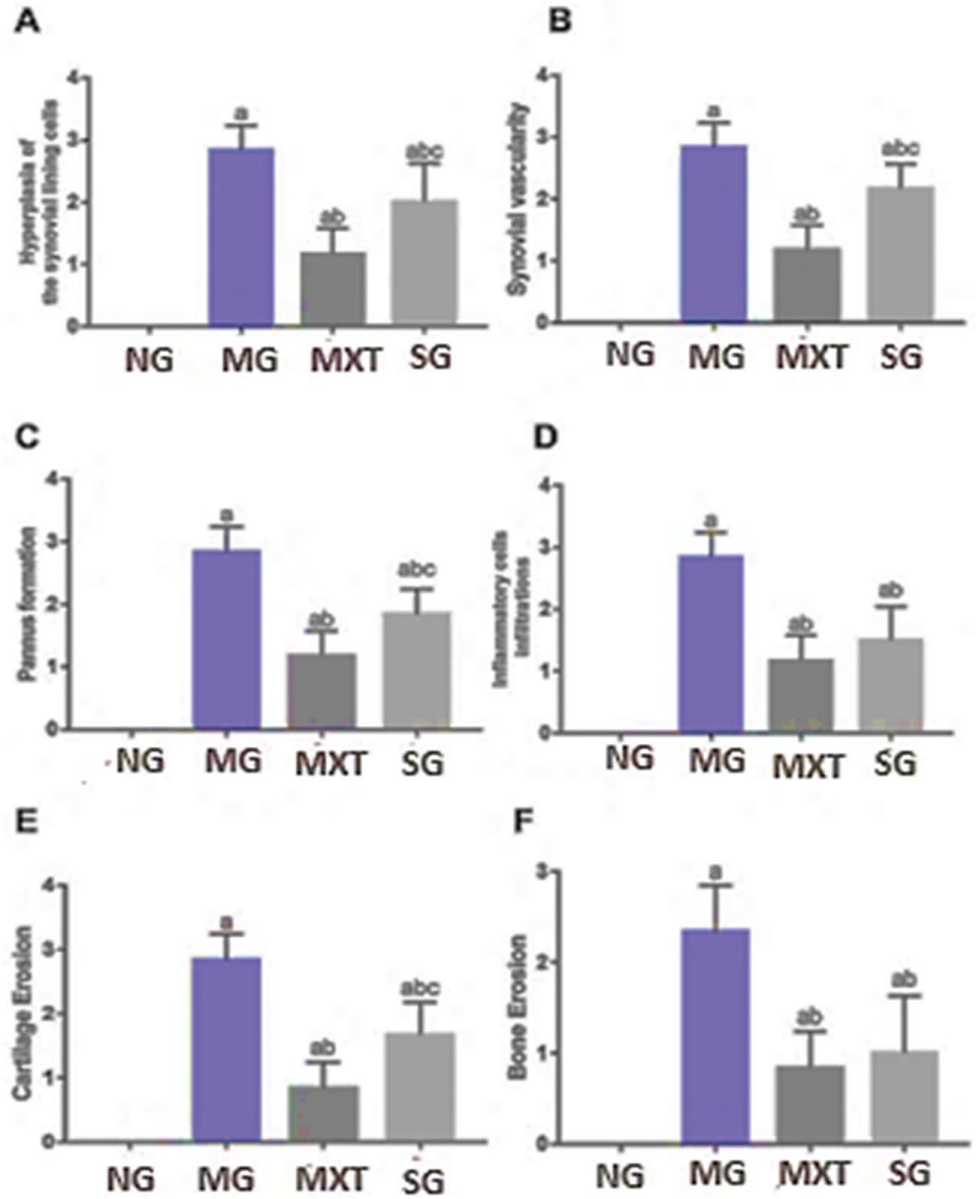
Semi-quantitative analysis of histopathological changes in the joints of arthritic rats. NG, normal group; MG, RA model group; MTX, methotrexate-treated rats; SG, *Spirulina* extract-treated group. Bar graphs represent mean ± SD (*n* = 6), with letters indicating significant differences compared with the normal rats (a), RA rats (b), and MTX rats (c) at p ≤ 0.05 using One way ANOVA followed by Tukey's test for post-hoc analysis

In comparison with the RA-induced arthritic group, these histopathological symptoms were significantly mitigated after treatment with MTX, with only mild morphological changes in the ankle, minimal inflammatory cells infiltrations, reduced synovial hyperplasia, and limited cartilage and bone erosion observed (Fig. 2C). On the other hand, *S. platensis*-treated rats showed moderate improvement in these lesions, with moderate inflammatory cells infiltrations and reduced synovial hyperplasia, cartilage destruction, and bone erosion (Fig. 2D). Correspondingly, the RA group’s histopathological scores rose significantly compared with the control rats (Fig. 3). The MTX-treated groups showed a substantial decrease in lesion score compared to the RA group (Fig. 3). In contrast to the RA group, *S. platensis*-treated groups showed a slight decline in lesion scoring (Fig. 3).

### Metabolomics study of serum samples

RA is a multilevel autoimmune disorder characterized by chronic synovial inflammation and progressive cartilage destruction in the absence of appropriate treatment (Giannini et al. 2020). Compelling reports have shown that probing mechanisms are essential for early diagnosis and treatment efficacy evaluations (Bartikoski et al. 2022). In line with this, serum metabolomic analysis can offer an association study of metabolic patterns and marker metabolites and, therefore, reflect the body’s functioning state and create a bio-evaluation system for medication effectiveness, opening the door for the creation of new therapeutic alternatives (Zhang et al. 2012). While *S. platensis* has been increasingly used in the prevention and treatment of chronic autoimmune diseases (Khan et al. 2005), its specific efficacy in RA and the underlying mechanisms remain unclear.

### Metabolic profile and pattern recognition of serum samples

**Fig. 4:**
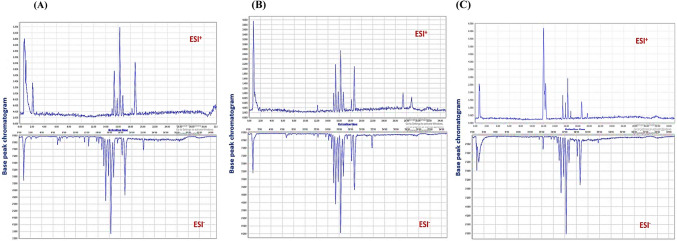
Representative UPLC-MS base peak chromatograms (BPCs) collected from serum samples of the normal group (**A**), RA model group (**B**), and *S. platensis* group (**C**), in both positive and negative electrospray ionization modes, respectively

With a view to systematically demonstrating the mechanisms behind *S. platensis’s* ability to alleviate RA, UPLC-MS/MS was successfully conducted to analyze all serum samples in both positive and negative ionization modes, affording a thorough view of a large variety of metabolites. Representative base peak chromatograms (BPCs) of serum samples from the control, model, and *Spirulina* groups were obtained under proper conditions (Fig. 4). Details regarding the detected metabolites’ peak areas are given in the Supplementary material, Table S4. To ensure the repeatability and stability of the analytical system, the RSDs of the retention times for five selected ions were calculated as 0.12–0.25 and 0.10–0.53% in positive mode and 0.06–0.80 and 0.08–0.78% in negative mode, respectively. The RSDs of peak areas were 0.63–8.53 and 0.85–10.03% in positive mode and 1.98–10.88 and 4.67–11.79% in negative mode, respectively, indicating that the established UPLC-MS/MS method exhibited good repeatability and stability and that the obtained data could be used for subsequent metabolomics research.

PCA, a statistical technique for dimensionality reduction, is dominantly applied for variable averaging and categorization from an unbiased viewpoint. Coincidentally, the PCA model defined by five components (Fig. S3) successfully discriminated the three groups—normal, model, and *Spirulina*—into two major clusters along the first two orthogonal PCs, explaining 77.3% of the total variance in which the CFA model group was far away from the normal group, suggesting the successful establishment of the RA model revealing significant differences in serum endogenous metabolites. The *Spirulina* group and the normal group were comparatively close together on the positive side of PC1, preliminarily demonstrating that *Spirulina* exerted a positive regulatory trend on the plasma metabolite-related RA.

As a mode recognition method, OPLS-DA, affording another classification attempt directed by well-established PCA scores, was conducted to maximally emphasize the logical distinctions between the control group, RA group, and *Spirulina* group, succinctly highlighting the metabolites with significant discrepancy among the groups.

**Fig. 5:**
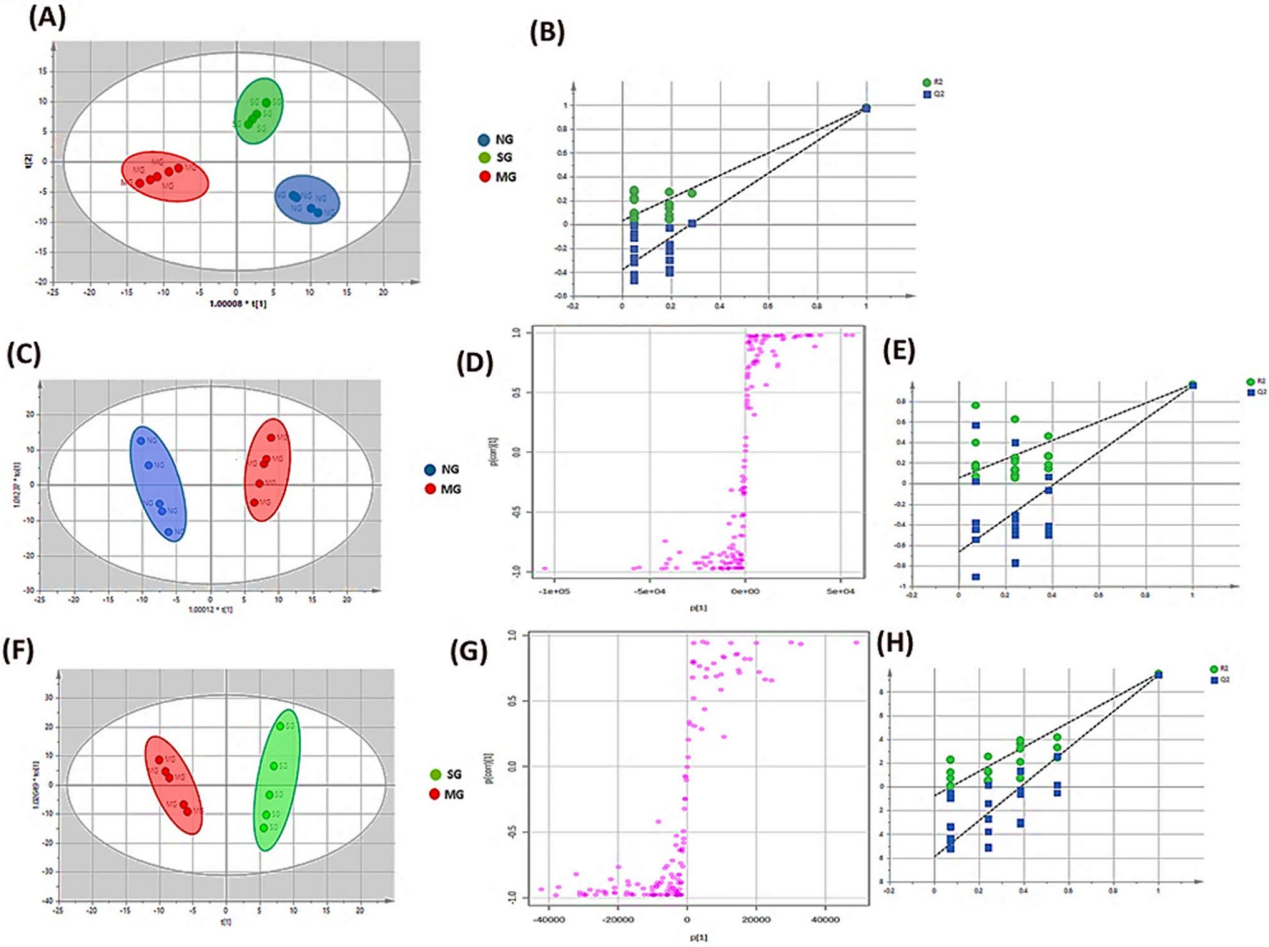
**A** OPLS-DA score plot of the three groups based on the plasma metabolic profiles. **B** The corresponding permutation test (*n* = 20 iterations) for the OPLS-DA model. **C**–**E** OPLS-DA score plots of the normal and model groups, the corresponding S-plot, and permutation test (*n* = 20 iterations) respectively. **F**–**H** OPLS-DA score plot of *Spirulina* and model groups, the corresponding S-plot, and permutation test (*n* = 20 iterations), respectively

As presented in Fig. 5A, the OPLS-DA score chart of the three groups showed a clear separation tendency, with the model group grouping in the opposite way from the control and *Spirulina* groups, inferring statistical biochemical disturbances existed in these groups. Coincidentally, the *Spirulina* group further deviated from the model group and was considerably closer to the control group, suggesting compelling proof that *S. platensis*, equipped with a variety of weaponry to regain the harmony disrupted in the RA state. Correspondingly, the OPLS-DA diagram in Fig. 5C showed that there was distinguishable discrimination between the normal and model groups, inferring that model induction altered the serum metabolic patterns in normal rats. Given this connection, as shown in Fig. 5F, an obvious separation was noted between the model and *Spirulina* groups in the OPLS-DA score plot, suggesting that *Spirulina* can reverse the pathological state in arthritic rats, shifting the disordered endogenous metabolites to the normal-like level.

Metabolites that met the screening criteria of VIP > 1, FC > 2.0 (or < 0.5), and *p* values < 0.05 were shown to be significantly relevant for clustering and discrimination. As seen in Fig. 5D, G, the respective S-plots of control versus model group and model versus treated were constructed to pick out the potential biomarkers where the ions farther away from the origin exhibited high VIP values and contributed dominantly to the discrimination between each of the two groups. To judge the performance of OPLS-DA models, the calculation of the *R*_2_*Y* (explained variance) and *Q*^2^ (predicted variance) parameters approached 1.0, affirming the reliability and predictive ability of the created models. In addition, the permutation tests (*n* = 20) showed that the OPLS-DA models were not over-fitted, contrasting that the established OPLS-DA models had prominent fitness and better predictability (Fig. 5B, E, H).

### Identification of potential endogenous biomarkers

**Fig. 6:**
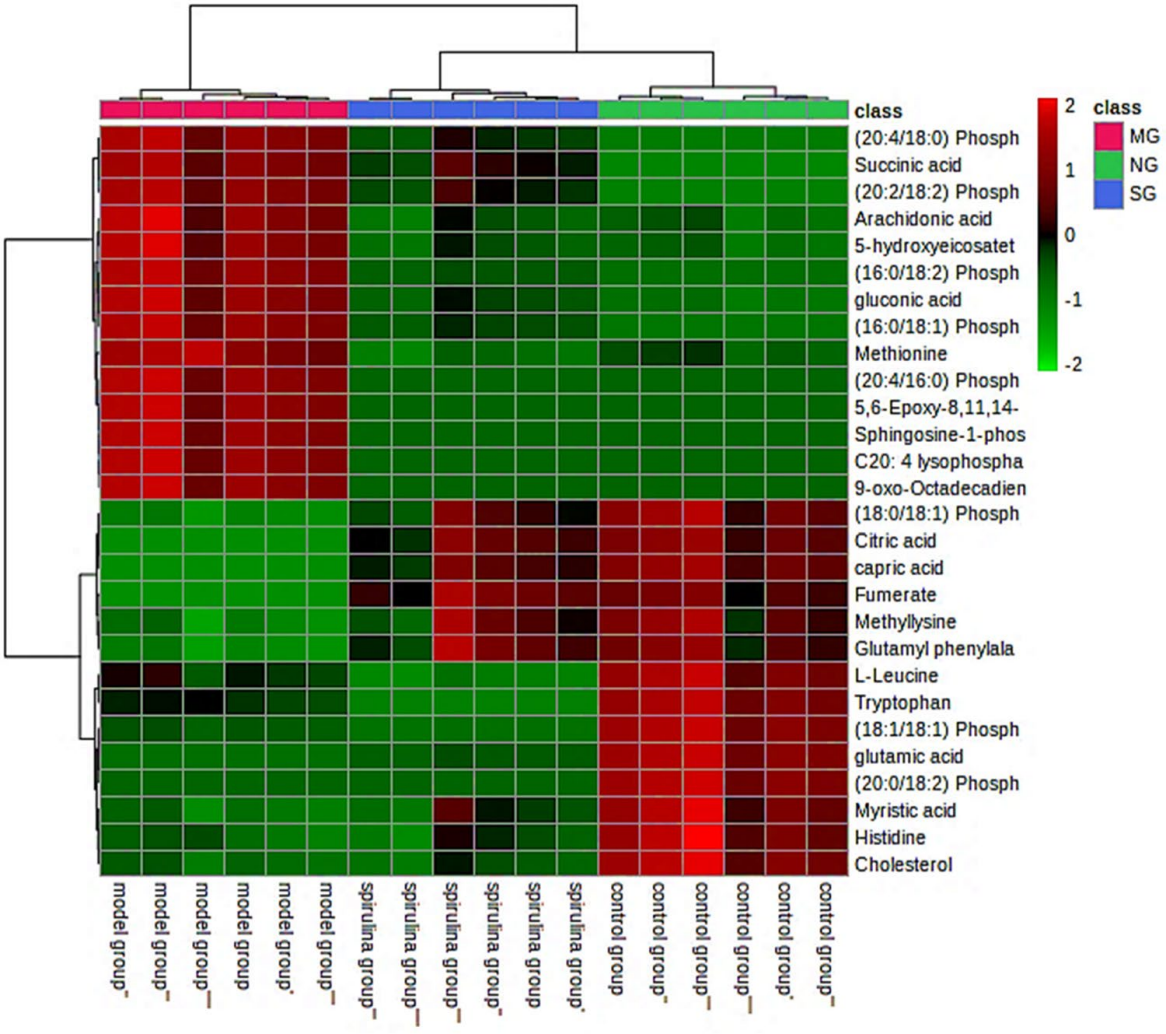
Heat map showing differential metabolite content in serum samples from the normal group (NG), arthritic model group (MG), and *Spirulina* group (SG). Note: Red represents increased content, while green indicates decreased content

Consistent with the criteria above, the unique metabolic features that significantly contribute to the grouping and discrimination were further identified by aligning the mass data (m/z) with the metabolomics databases HMDB and METLIN. The potential biomarkers were further verified by mass data of the distinctive ions and fragmentation patterns. Table 3 shows the identified biomarkers of RA and their metabolic pathways. As indicated by the results, compared with the control group, a total of 28 RA-related endogenous metabolites (14 down- and 14 upregulated according to the normalized abundance comparison) spanning varying classes as amino acids, fatty acids and their derivatives, and sugars with significant amounts of lipid species, including phosphatidylcholines (PCs), phosphatidic acids (PAs), and sphingolipids, were tentatively assigned through mass spectrometry’s full scanning mode, and their changes among different groups are shown in Table 3. Meanwhile, after the *Spirulina* intervention, 20 biomarkers (*versus* the model group) tended to return to normal levels or showed significant reversing tendencies, ascertaining their potential correlativity and therapeutic worth for the control of RA, as discussed later. Furthermore, regarding the expression abundance of each potential biomarker, a heatmap was established to trace the dynamic metabolic events among rat groups. The heatmap (Fig. 6) displayed the overview alterations in the serum metabolome of different groups where metabolites with comparable abundance fluctuation tendencies were grouped. The heatmap heightened the close grouping of the metabolites in the *Spirulina* group and their distinction from the MG group, convincingly indicating that *Spirulina* could modulate the endogenous metabolites disturbed by RA.

**Table 3:** Differentially expressed endogenous metabolites in the serum profile, their change trends among groups, and the altered metabolic pathways in RA

No	RT/min	Metabolites	m/z	Formula	Trend NG/MG	Trend MG/SG	MS/MS fragments	Related pathway	HMDB ID
					VIP–FC–*p* value	VIP–FC–*p* value			
1	0.65	Citric acid*	191.2 [M − H]^−^	C_6_H_8_O_7_	1.45–6.62–6.6841*E* − 7	1.43–0.16–0.001	173–111	Citrate cycle (TCA cycle)	HMDB0000094
2	0.77	Fumarate	113.1 [M − H]^−^	C_4_H_2_O_4_^−2^	1.43–6.4–6.6767*E* − 7	1.4–0.16–0.001	85	Citrate cycle (TCA cycle)	HMDB0000134
3	0.92	Gluconic acid	195.2 [M − H]^−^	C_6_H_12_O_7_	1.26–0.24–8.7835*E* − 7	1.31–5.2–0.002	109–99	Glycolysis/gluconeogenesis	HMDB0000625
4	1.2	Succinic acid	117.1 [M − H]^−^	C_4_H_6_O_4_	1.33–0.24–1.5449*E* − 7	1.28–2.05–0.005	89	Citrate cycle (TCA cycle)	HMDB0000254
5	1.6	Glutamic acid^b^	146.1 [M − H]^−^	C_5_H_9_NO_4_	1.45–6.6–6.6855*E* − 7	1.4–0.15–0.001	132	d-Glutamine and d-glutamate metabolism	HMDB0000148
6	1.84	Histidine	156.2 [M + H]^+^	C_6_H_9_N_3_O_2_	1.2–2.3–1.1639*E* − 4	–^a^	128–111	Histidine metabolism	HMDB0000177
7	1.95	l-Leucine	132.2 [M + H]^+^	C_6_H_13_NO_2_	1.28–2.02–1.1189*E* − 4	–^a^	104–87	Valine, leucine, and isoleucine degradation	HMDB0000267
8	2.17	Methionine	172.3 [M + Na]^+^	C_5_H_11_NO_2_S	1.33–0.47–3.9098*E* − 6	1.27–2–0.001	104	Cysteine and methionine metabolism	HMDB0000696
9	4.3	Methyllysine	183.2 [M + Na]^+^	C_7_H_16_N_2_O_2_Na	1.28–2.05–1.1189*E* − 4	–^a^	147–130–84	Valine, leucine, and isoleucine degradation	HMDB0002038
10	5.17	Tryptophan	205.1 [M + H]^+^	C_11_H_12_N_2_O_2_	1.36–2.3–1.1389*E* − 4	–^a^	188–144	Tryptophan metabolism	HMDB0013609
11	10.24	Glutamyl phenylalanine	293.2 [M − H]^−^	C_14_H_18_N_2_O_5_	1.3–2.4–3.1689*E* − 5	1.26–0.49–0.004	166–145–102	d-Glutamine and d-glutamate metabolism	HMDB0000594
12	11.3	Capric acid	172.3 [M + H]^+^	C_10_H_20_O_2_	1.4–6.2–6.6841*E* − 7	1.4–0.18–0.001	143	Fatty acid biosynthesis	HMDB0000511
13	13.6	Cholesterol	407.4 [M + Na]^−^	C_27_H_46_O	1.27–2.7–1.1689*E* − 4	–^a^	367–345	Steroid hormone biosynthesis	HMDB0000067
14	14.22	Myristic acid	265.2 [M + K]^−^	C_14_H_28_O_2_	1.28–2.5–3.7852*E* − 5	1.22–0.58–0.002	209	Fatty acid biosynthesis	HMDB0000806
15	15.4	Sphingosine- 1-phosphate	378.2 [M − H]^−^	C_18_H_38_NO_5_P	1.3–0.17–4.9246*E* − 7	1.27–6.2–0.001	301–105–88	Sphingolipid metabolism	HMDB0000277
16	16.8	C20: 4 lysophosphatidylinositol	619.3 [M − H]^−^	C_29_H_49_O_12_P	1.4–0.16–4.9246*E* − 7	1.36–6.2–0.0010	303	Glycerophospholipid metabolism	HMDB0254272
17	20.32	(18:1/18:1) Phosphatidic acid	699.6 [M − H]^−^	C_39_H_72_O_8_P	1.32–5.2–1.0332*E* − 6	–^a^	418	Glycerophospholipid metabolism	HMDB0007865
18	21.3	5-Hydroxyeicosatetraenoic (5-HETE)	319.2 [M − H]^−^	C_21_H_34_O_2_	1.3–0.33–7.1716*E* − 6	1.29–3.3–0.002	303–285–259	Arachidonic acid metabolism	HMDB0011134
19	22.19	(18:1/18:0) Phosphatidic acid	701.6 [M − H]^−^	C_39_H_74_O_8_P	1.3–3.4–6.1658*E* − 6	1.27–0.43–0.005	446–416	Glycerophospholipid metabolism	HMDB0008303
20	22.9	(16:0/18:2) Phosphatidylcholine	758.2 [M + H]^+^	C_42_H_80_NO_8_P	1.34–0.16–4.9246*E*− 7	1.28–2.4–0.001	503–479	Glycerophospholipid metabolism	HMDB0007973
21	23.2	9-oxo-Octadecadienoic acid	295.3 [M + H]^+^	C_19_H_34_O_2_	1.4–0.16–4.9246*E* − 7	1.4–6.2–0.001	279–251	Biosynthesis of unsaturated fatty acids	HMDB0004669
22	24.18	(16:0/18:1) Phosphatidylcholine	804.5 [M + HCOO^−^]^−^	C_43_H_83_NO_10_P	1.4–0.16–4.9277*E* − 7	1.32–3.5–0.005	523–549	Glycerophospholipid metabolism	HMDB0007971
23	24.8	(20:4/16:0) Phosphatidylcholine	782.6 [M + H]^+^	C_44_H_80_NO_8_P	1.34–0.16–4.9365*E* − 7	1.32–6.2–0.001	527–471	Glycerophospholipid metabolism	HMDB0007982
24	26.8	Arachidonic acid^b^	325.2 [M + Na]^−^	C_20_H_32_O_2_	1.3–0.42–2.4599*E* − 5	1.28–2.5–0.003	285–259	Biosynthesis of unsaturated fatty acids	HMDB0001043
25	28.6	5,6-Epoxy- 8,11,14-eicosatrienoic acid	343.3 [M + Na]^+^	C_20_H_32_O_3_	1.32–0.16–4.9246*E* − 7	1.3–6.2–0.001	276	Arachidonic acid metabolism	HMDB0002190
26	29.12	(20:4/18:0) Phosphatidylcholine	810.3 [M + H]^+^	C_46_H_84_NO_8_P	1.4–0.16–4.9308*E* − 7	1.28–2.5–0.004	527	Glycerophospholipid metabolism	HMDB0008048
27	29.8	(20:2/18:2) Phosphatidylcholine	854.5 [M + HCOO^−^]^−^	C_46_H_84_NO_8_P	1.4–0.16–4.9246*E* − 7	–^a^	575–547	Glycerophospholipid metabolism	HMDB0008336
28	30.62	(20:0/18:2) Phosphatidylserine	815.6 [M − H]^−^	C_44_H_82_NO_10_P	1.42–6.1–5.9246*E* − 7	–^a^	536	Glycerophospholipid metabolism	HMDB0010164

*MG*, model group; *NG*, control group; *SG*, *S. platensis* group; *VIP*. variable importance in the projection; *RT* (min), retention time; *FC*, fold change (as determined by average relative quantitation obtained from group 1/group 2, and a value less than 1 indicates a decline in the metabolites of group 1)

^a^The corresponding metabolite did not fulfill the screening criteria

^b^Identified by comparison with authentic references

### Metabolic pathway analysis

**Fig. 7:**
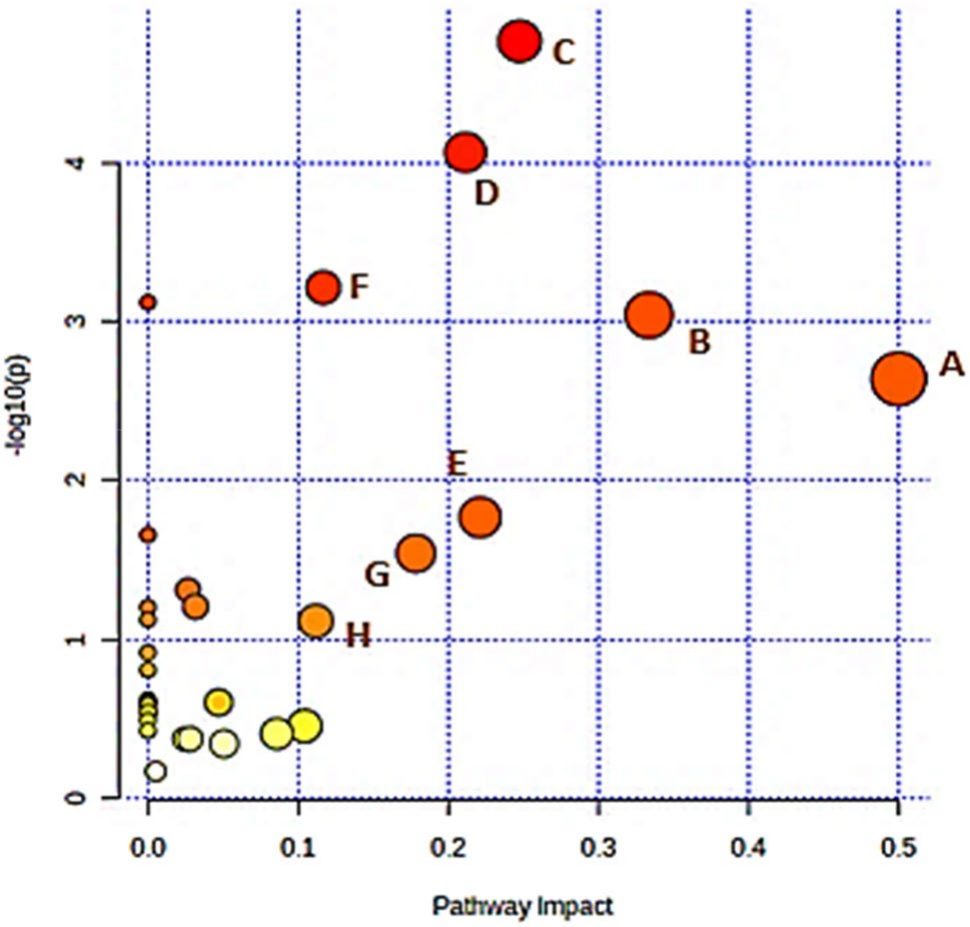
Summary of metabolic pathway analysis for differential metabolites involved in RA. **A** d-Glutamine and d-glutamate metabolism; **B** arachidonic acid metabolism; **C** alanine, aspartate, and glutamate metabolism; **D** citrate cycle (TCA cycle); **E** histidine metabolism; **F** arginine biosynthesis, **G** sphingolipid metabolism; and **H** glycerophospholipid metabolism

The HMDB ID of these putative biomarkers was imported into MetaboAnalyst 5.0 to generate pathway analysis and enrichment overview analysis establishing a link between RA and metabolic profiles and fully clarifying regulative mechanisms through which *Spirulina* mitigates against RA in a holistic fashion, and the results are presented in the form of a bubble diagram (Fig. 7). The endogenous differentials were found to be connected with 28 metabolic pathways disordered in the model group inferring that RA is a complicated metabolic illness brought on by several interrelated variables, and the impact of several metabolic pathways was evaluated by topology map generated from MetaboAnalyst 5.0 (Fig. 7; Table S5). By setting raw *p* < 0.05 and impact value > 0.1 as the screening threshold, we filtered out eight potential metabolic pathways essentially implicated in lipid metabolism, amino acid metabolism, energy, glycometabolism, and gluconeogenesis. As shown in Fig. 7, the most related pathways, namely d-glutamine and d-glutamate metabolism, arachidonic acid metabolism, alanine, aspartate and glutamate metabolism, citrate cycle (TCA cycle), histidine metabolism**,** arginine biosynthesis, sphingolipid metabolism, and glycerophospholipid metabolism, are highlighted. These results revealed the complex pathological mechanism involved in RA and the pharmacological mechanism of *Spirulina* action. Essentially, each of these pathways exhibits a distinct function in immune cells and is controlled to meet biological needs.

Relatedly, to better understand the underlying pathophysiology of RA, differential endogenous metabolites were categorized into three primary groups according to their biochemical functions (based on the KEGG and HMDB databases): (1) amino acid metabolism primarily affected by l-leucine, methionine, glutamic acid, and methyl lysine; (2) lipid metabolism most enriched with arachidonic acid, capric acid, sphingosine- 1-phosphate, 5-hydroxyeicosatetraenoic, phosphatidylcholines (PCs), and phosphatidic acids (PAs); and (3) energy, glycol-metabolism, and gluconeogenesis metabolism was implicated by metabolites; citric acid, succinic acid, fumarate and gluconic acid. The results of the serum metabolomic analysis are covered in detail in the ensuing subsections.

### Amino acid metabolism

Accumulating evidence has shown that amino acid metabolism is principally involved in immunity, inflammation, and the nervous system (Duarte-Delgado et al. 2022). Herein, amino acid metabolism in RA model rats was disordered, revealing decreased relative levels of leucine, methyl lysine, glutamic acid, and tryptophan, while methionine revealed a tendency for up-regulation in the arthritic group. This tendency is positively consistent with several lines of evidence that demonstrated the primary disrupted trend for amino acid levels in the RA model is a decline, suggesting that the protein is broken down into amino acids in response to inflammation, energy expenditure, and autoimmune responses (Duarte-Delgado et al. 2022). Importantly, a key pathogenic characteristic of RA is the hypoxic synovial tissue microenvironment, which severely aggravates joint inflammation and tissue destruction, and increases energy expenditure (Giannini et al. 2020). More specifically, the homeostasis of energy metabolism and amino acid synthesis may be directly impacted by RA.

In this study, glutamic acid level exhibited a clear downregulating trend compared with normal rats as glutamic acid has been associated with RA-related target genes located in the nucleus, cytoplasm, and extracellular space and participates in inflammatory response, organismal injury, and skeletal and muscular disorders, aggravating the empirical pathological state in the body (Kelley et al. 2008).

In this context, lower serum concentration of glutamyl phenylalanine interrelated to altered glutamate metabolism was detected in MG rats. Relatedly, a leading study pointed out that chondrocytes utilize glutamic acid for energy release in an anabolic state in RA. Deprivation of glutamic acid itself causes metabolic reprogramming and reduces chondrocyte inflammatory response by inhibiting NF-κB activity (Arra et al. 2022). Coincidentally, glutamic acid is primarily involved in the glutathione cycle, serving as a core marker for mitigating oxidative stress in RA (Arra et al. 2022), which is in accordance with biochemical results.

In contrast, a rise of methionine content in MG rats compared to the matched controls is intimately connected with the pathologic process of immunological disorders in RA as acute arthritic conditions, oxidative burst, and neutrophil activation under RA state where methionine has been linked with a handful of physiological events with a special impact on maintaining fuel homeostasis in the human body and regulating differentiation and functional expression of T cells in times of inflammation and got a back-regulated after treatment with *Spirulina* extract (Shi et al. 2022).

In short, these metabolic disruptions in amino acid metabolism can give the energy needed in the pathogenic setting of RA and supply the building blocks for the proliferation of immunological and synovial cells. Thus, they may be attractive candidates for tracking RA disease activity and possible RA treatment options for *Spirulina* (Panfili et al. 2020). Importantly, under the premise of therapeutic efficacy, compared with the control group, with *Spirulina* administration, the average level of putative indicators linked to amino acid metabolism was reversed to varied degrees.

### Lipid metabolism

Importantly, lipid metabolism plays central overlapping roles in all aspects of biological functions, principally including cell proliferation, immunity, angiogenesis, and inflammation (Luan et al. 2021). According to some scholars, RA’s lipid components are typically harmful to the body, and disruptions in lipid metabolism have been linked to metabolic signaling pathways or inflammatory/immune activity, which may have contributed directly or indirectly to the development, progression, and recurrence of RA (Duarte-Delgado et al. 2022). In our investigation, we found that the lipid metabolism of RA rats was aberrant.

All mammalian cell membranes are mostly composed of phospholipids, which are also the synthetic building blocks of lipoproteins and participate in a variety of metabolic and signal transduction processes (Calvano et al. 2020). Abnormalities in glycerophospholipid metabolism can promote systemic inflammation and immune activation, which are considered clear risk factors for RA (Duarte-Delgado et al. 2022). In the present research, phosphatidylcholines (PCs), phosphatidic acids (PAs), and lysophosphatidylinositols (LysoPIs) were observed to be the key phospholipids implicated in the glycerophospholipid metabolic pathway and are estimated as biomarkers markedly perturbed in a variety of immune diseases in recent scholars (Xu et al. 2022). Accordingly, serum levels of PAs detected went down in the RA group, supporting their roles in inflammation and oxidative stress where PAs are implicated in diverse biological aspects, primarily the enzyme NADPH oxidase activation, which operates the defense mechanism against tissue damage in times of inflammation (Zhang et al. 2012). While C20: 4 lysophosphatidylinositol temporally synthesized by inflammatory stimuli exhibited adverse association where it externalizes at the sites of inflammation as a chemoattractant promoting cell migration and inflammatory events (Buhrmann et al. 2011). In parallel, PC subclasses namely (16:0/18:2) phosphatidylcholine, (16:0/18:1) phosphatidylcholine, (20:4/18:0) phosphatidylcholine, and (20:4/16:0) phosphatidylcholine levels were overexpressed in the RA group where they act as signaling molecules modulating cellular and tissue adaptation to inflammatory cascades (Luan et al. 2021). Following *Spirulina* intervention, the levels of these phospholipid metabolites in RA rats’ serum tended to be normal.

In this investigation, an elevated level of arachidonic acid was found in RA rats, which is primarily tied to the imbalance of arachidonic acid metabolism in the body under RA state. This pattern is consistent with data that has been previously published (Ye et al. 2021). Arachidonic acid (AA) is a physiologically active n- 6 polyunsaturated fatty acid, produced from phospholipids in reaction to oxidative stress (Wada et al. 2007). Under cyclooxygenases'catalysis, AA was subsequently converted into multifarious inflammatory active mediators, namely prostaglandins (PGs), leukotrienes, and lipoxins. These metabolic regulators in turn play an indispensable role in immunity, synovial hyperplasia, inflammation, and cartilage damage of RA (Meng et al. 2015). Furthermore, the upregulation of AA’s cyclooxygenase and lipoxygenase pathways is tightly connected with the increased generation of reactive oxygen species (ROS) and reactive nitrogen species (RNS) in reaction to inflammation and RA development (Ferreira et al. 2021). Worth noting, these reports came in good harmony with oxidative and inflammatory markers, which witnessed a significant elevation in the RA group but were adjusted to normal levels after *Spirulina intervention* through modulating the disturbed arachidonic acid metabolism pathway.

Another noteworthy finding related to the above is the significant elevation of 5-hydroxyeicosatetraenoic (5-HETE) acid, a pro-inflammatory eicosanoid from AA, in the model group, which consequently modulates inflammatory response and induces the expression of MMP2 and MMP9 to promote angiogenesis (Xu et al. 2022). Furthermore, there were other inflammatory lipid mediators related to alterations in arachidonic acid metabolism and abnormally elevated in the model group like 5,6-epoxy- 8,11,14-eicosatrienoic acid (EET), which possess vasodilator and natriuretic effects. These results were probably connected to the onset of acute inflammation and oxidative stress in the pathological state of RA. Certainly, the arachidonic acid metabolic pathway is complex and directly linked to RA-related inflammatory response, pannus formation, joint pain and swelling as well as cartilage and bone destruction (Meng et al. 2015). Remarkably, *Spirulina* exhibited positive adjustment effects on AA, 5-HETE, and EET levels. A similar up-regulation trend of 9-oxo-octadecadienoic acid was found in the model group, which putatively implicated in mediating inflammatory signaling pathways by modulating immune cell differentiation and trafficking (Dawczynski et al. 2009). Meanwhile, *Spirulina* showed significant regulative efficacy on 9-oxo-octadecadienoic acid to control like levels. Conversely, capric and myristic acid levels were significantly depressed in the model rats compared to the normal group, inferring that the fatty acid biosynthesis pathway in the RA group was perturbed, reflecting a state of dampened energy generation, elevation of innate immune cytokines, and high oxidative stress (Blackmore et al. 2020). According to our research, supplementing with *Spirulina* could successfully halt this downward trend.

Additionally, one of the major metabolic pathways that can be altered in RA rats was associated with sphingolipid metabolism. As the data shows, higher serum level of sphingosine 1-phosphate (S1P) was detected in RA rats compared with the control ones, indicating impaired sphingolipid metabolism, which was consistent with a previous report (Hu et al. 2011). Sphingolipids are essential molecules of cell membranes affecting a variety of regulatory biological processes, including cell migration, differentiation, and apoptosis, orchestrating inflammation and immune surveillance (Hannun and Obeid 2018). When sphingolipid metabolism is dysregulated, its downstream S1P, one of the bioactive metabolites of sphingolipid, could initiate a variety of signaling pathways that lead to the release of pro-inflammatory cytokines as IL- 2 and IL- 6, exacerbating inflammatory processes (Hu et al. 2011). Equally important, sphingolipid metabolism and oxidative stress are complexly related, where sphingolipids act as second messengers to increase oxidant production (Ferreira et al. 2021). Therefore, the targeted knockout of crucial elevated metabolites can show potential roles in RA control. Abnormal sphingolipid metabolism during the RA state negatively affects liver function and regeneration, concluding with liver fibrosis typically manifested with a rise in the liver enzyme (AST and ALT) (Nojima et al. 2024). Interestingly, these investigations are consistent with the current biochemical markers’ findings. Worthily, the two RA-associated metabolites above had a callback tendency to a normal-like state after *Spirulina* administration.

### Energy, glycometabolism, and gluconeogenesis metabolism

As alluded to above, rising energy demand, which is vital for immune cell development, proliferation, and speeding up pro-inflammatory chemical flow, is a hallmark of persistent inflammation and immunological activation (Bartikoski et al. 2022). It has been reported that systemic inflammation brought on by RA can speed up energy metabolism (Zhang et al. 2012). Correspondingly, the tricarboxylic acid (TCA) cycle is the axis that links amino acid, fatty acid, and glycolysis metabolism (Duarte-Delgado et al. 2022). Certainly, it is the central metabolic pathway for energy flow in the body incorporated in adenosine triphosphate (ATP) synthesis (Duarte-Delgado et al. 2022). Our study evidently highlighted perturbations in glycolysis metabolism as well as the TCA cycle, and these changes are reflected in four pivotal metabolites: citric acid, gluconic acid, fumarate, and succinic acid. Herein, a sharp drop of citric acid in the RA group compared with healthy controls was observed, indicating the restricted function of the TCA cycle consistently with a universal characteristic of the inflammatory state in the pathogenesis of RA. Likewise, fumarate was significantly depressed in the RA group. In the TCA cycle, fumarate is converted to maleate by the enzyme fumarase to control fuel homeostasis in the human body in the form of ATP production, orchestrate the migration and proliferation of epithelial cells, and spatiotemporally regulate differentiation and functional expression of T cells (Moharregh-Khiabani et al. 2009). Also, during impaired mitochondrial function in the RA state, citric acid and its metabolites, mainly fumaric acid, regulate the production of inflammatory cytokine and ROS in arthritic joints. These records were in good alignment with the current observations.

In alignment with previous publications (Li et al. 2018), an abnormal increase of succinic acid in the RA group was observed. Succinate accumulation can mediate immune cell activation and maintain IL- 1β production prevalent in inflammatory environments (Blagov et al. 2022). Equally important, glucose metabolism via glycolysis allows cells to generate ATP principles for immunity and inflammatory pathway activation under hypoxia conditions (Garcia-Carbonell et al. 2016). Consistent with earlier records (Shi et al. 2022), there was a noticeable rise in the glucose derivative gluconic acid, as a collateral consequence of mitochondrial ultrastructure damage and amplified energy demand under hypoxia conditions associated with the exacerbated RA symptoms. Interestingly, *Spirulina* intake resulted in favorable recovery performance of these disordered biomarkers toward normal levels.

In light of the above findings, we concluded that *S. platensis* extract enriched with different biologically active compounds, primarily flavonoids, fatty acids, phlorotannins, and carotenoids could synergistically mitigate RA by normalizing oxidative stress, inflammation, and liver and kidney functions. Furthermore, this marine wealth could effectively restrain the disordered metabolites and their related pathways typically implicated in RA pathogenesis to normal-like.

Unfortunately, it is undeniable that the current incorporation of *Spirulina* products in the clinical trials against RA faces obstacles, primarily bioavailability, flowability, and sustained effect. However, *Spirulina* extract is nowadays delivered orally via a new double-coated layer tablet formulation to maximize bioavailability and delay the drug’s release from stomach segments by improving the early release hindrance of basic medications in acidic settings (ElFar et al. 2022). This formulation allows *Spirulina* to be delivered in femtoliposome carriers to the targeted sites for effective therapeutic outcomes (ElFar et al. 2022).

Even though the pharmacological mechanism of *Spirulina* against RA was expounded based on metabolic analysis, it necessitates further research to fully understand the mechanism from the upstream gene pathway to the downstream metabolic pathway. Genomics and proteomics analyses should be incorporated to precisely clarify the core genes and validate pathways associated with RA incidence and the *Spirulina* control mechanism.

Furthermore, global metabolic alterations in other biological fluids (such as urine and synovial fluid) should also be discussed to validate the biological roles of markedly changed with a scope to excavate the therapeutic mechanism through which *Spirulina* acts on RA in a holistic view, which is unquestionably very important in clinical settings.

## Conclusion

In summary, the control efficiency of *S. platensis* on RA was first traced in the current investigation through a series of RA-related biochemical and histopathological scores. *S. platensis* exerted profound therapeutic effects in the MG model by ameliorating paw edema and lowering systemic signs of inflammation. As the end of biological information flow, integration of the results of UPLC-MS-based serum metabolomics, the pattern recognition, along with metabolic pathway analysis has revealed high potential in profiling RA-specific metabolic features to systematically dissect the efficacy mechanism of *Spirulina* in RA mitigation. Overall, 28 differential endogenous metabolites in arthritis rats’ plasma primarily connected with the disturbance of arachidonic acid metabolism, alanine, aspartate, and glutamate metabolism, the citrate cycle (TCA cycle), and glycerophospholipid metabolism pathways were tentatively identified. Taking the potential biomarkers detected in the study as monitoring indexes, it revealed that *Spirulina* could effectively reverse the disrupted metabolites in RA rats to normal states by modulating the disturbed metabolic pathways.

All in all, our foundations combining serum metabolomics, metabolic pathway analysis, and experimental verification offered an innovative and workable guide for future pharmacological studies to support this study and fully uncover *S. platensis’s* fundamental therapeutic mechanism against RA on a systemic basis.

## Supplementary Information

Below is the link to the electronic supplementary material.

Supplementary file1 (DOCX 1004 KB)

## Data Availability

All source data for this work (or generated in this study) are available upon reasonable request.
