# Supplementary material for: Unveiling the pharmacological mechanisms of Spirulina platensis in rheumatoid arthritis rats through the integration of serum metabolomics, pathways analysis, and experimental validation
Source: Naunyn Schmiedebergs Arch Pharmacol. 2025 May 7;398(11):15495–513. doi: 10.1007/s00210-025-04191-y (PMC12552235; doi:10.1007/s00210-025-04191-y)
Supplement: Supplementary file 1 — Supplementary file1 (DOCX 1004 KB) [file 210_2025_4191_MOESM1_ESM.docx]

***Establishment of rheumatoid arthritis (RA) rats model and treatment***

All procedures in this study were approved by the Institutional Animal Care and Use Committee (IACUC), Alexandria university (Approval No: 0620228112123).

In a small-scale preliminary study, three doses of *S. platensis* extract were screened for anti-arthritic effects to convincingly select the optimal therapeutic dose of *S. platensis* extract being used in our recent investigation. These *Spirulina*-treated doses include low dose (200 mg/kg/day), medium dose (400 mg/kg/day) and high dose (800 mg/kg/day) (n = 3 per dose). In line with our preliminary observations regarding arthritis scores and previous studies investigating the optimal therapeutic dose of *S. platensis* in dose-responses approach [9, 10], it was found that *S. platensis* dose (400 mg/kg/day) effectively restrains the changes observed in CFA rats close to the normal direction as well as normalizes the joint histopathology of arthritic rats.

***Sample preparation and LC-MS parameters***

Accurate weight (1mg /mL) from lyophilized *Spirulina* extract was prepared in HPLC-grade methanol, ﬁltered through a 0.2 μm pore size membrane (Millipore) and degassed by sonication before injection. Full loop injection volume (10 μL) of the sample was applied onto the chromatographic column.

The binary mobile phase was comprised of acidiﬁed ultrapure water (0.1% formic acid) (Phase A) and acidiﬁed methanol (0.1% formic acid) (Phase B) was gradient eluted at a ﬂow rate of 0.2 mL min^−1^. The linear elution gradient program was stipulated as follows: 0% B –2 min, 2% B; 2–6 min, 2%–10% B; 6–14 min, 10%–28% B; 14–21 min, 28%–36% B; 21–25 min, 36%–55% B; 25–29 min, 55%–80% B; 29–33 min, 80%–100% followed by 5.0 min of post-run equilibration. The sample injection volume was set at 5 μL.

The suitably chosen set of operational parameters for ESI interface operated in both positive and negative ion modes with full scan function covering the mass range of 50–1200 Da were set as follows: capillary voltage of 3 kV, cone voltage; 35 V, the ion source temperature was 150◦C, the nebulizer (nitrogen gas) pressure was 35 psi, drying and sheath gas (N_2_) temperature was 440◦C and 350◦C, respectively. The drying and sheath gas ﬂows were applied at 900 L/h and 50 L/h, respectively. The scan time and interscan delay were set to 0.4 s and 0.1 s, respectively and the analytical run time was extended to 30 min. The MS/MS analysis was acquired in targeted MS/MS mode with 3 collision energies of 10 eV, 20 eV and 40 eV.


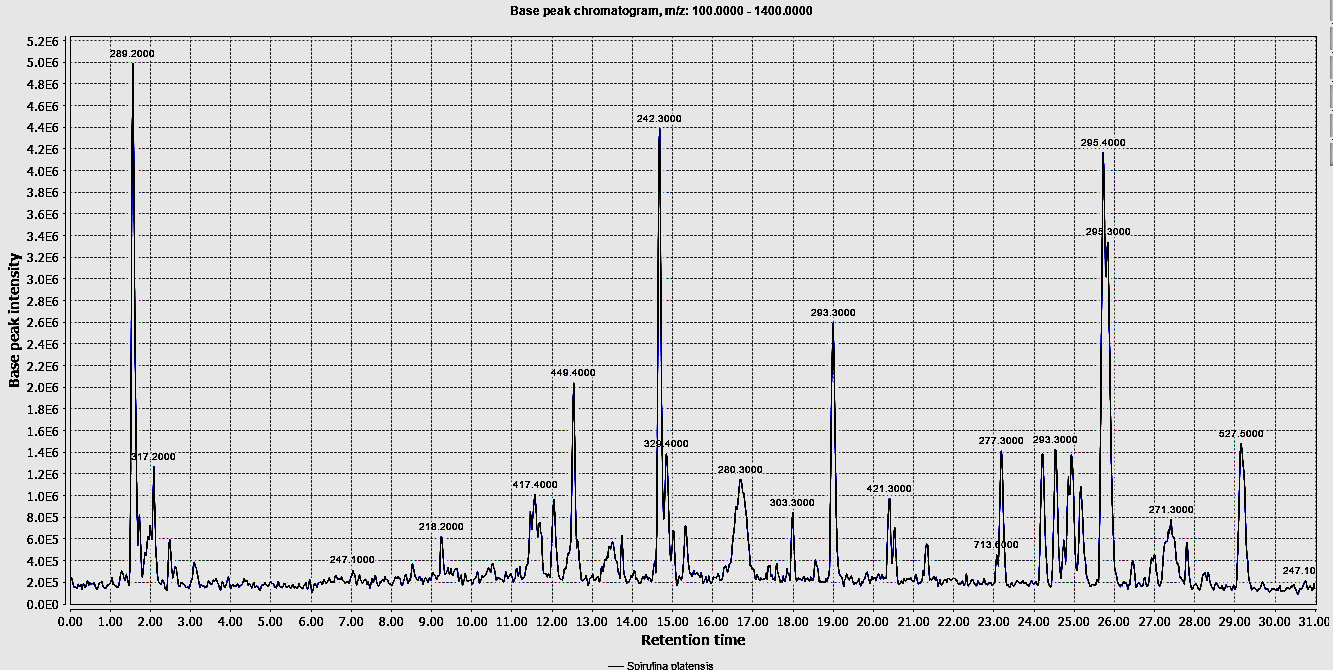


**B**

**A**


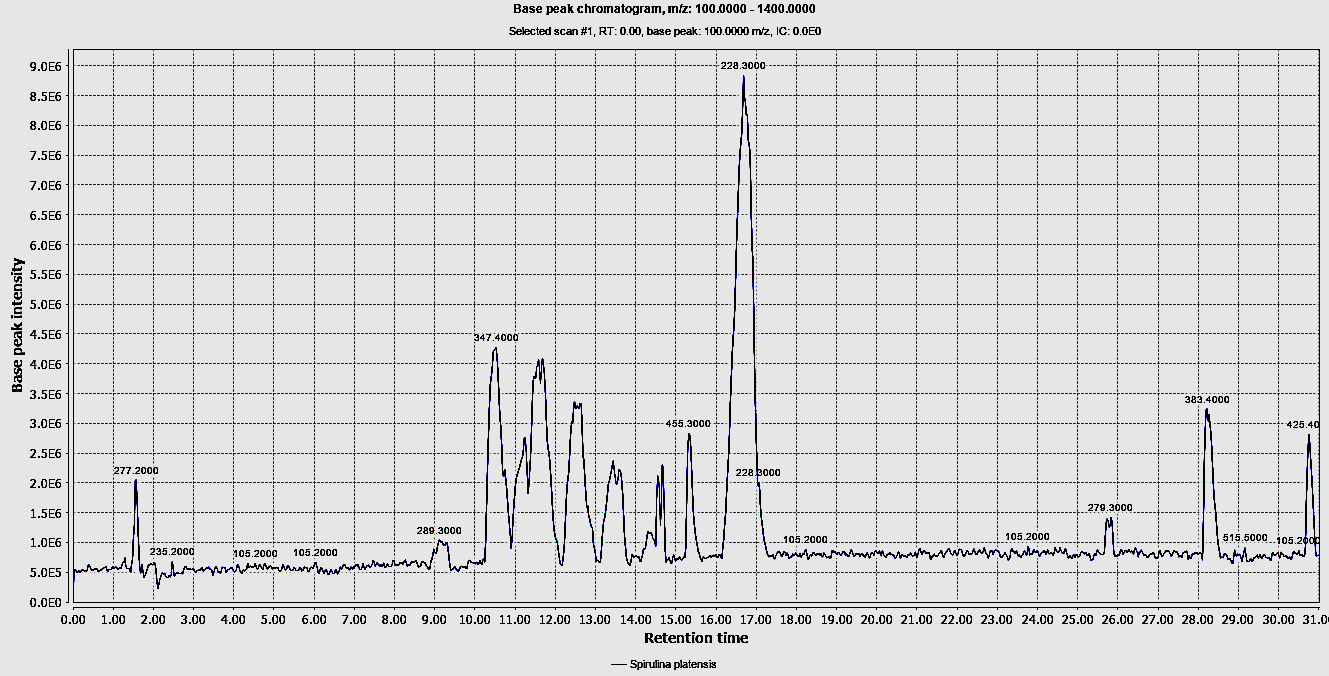


**Figure S1: Representative base peak chromatograms (BPC) collected from the extracts of (A / B) *Spirulina platensis* in both negative and positive ionization modes, respectively.**


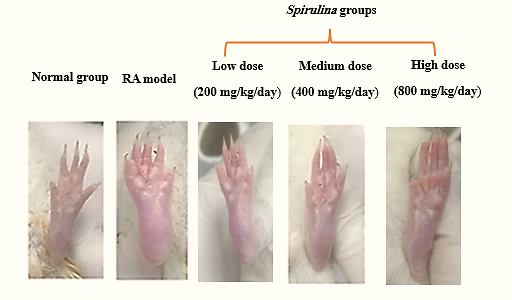


**Figure S2:** Representative photos showing the appearance of the right hind paw swelling of the rats in normal, RA model and *Spirulina*-treated doses including low dose (200 mg/kg/day), medium dose (400 mg/kg/day) and high dose (800 mg/kg/day). (n = 3 per dose).

**Table S1:** Details regarding age, gender and weight of each rat utilized in the current study

| **Rat No.** | **Rat Group** | **Rat body weight (gm)** | **Rat Gender** | **Rat Age (W)** |
| --- | --- | --- | --- | --- |
| 1 | Normal group | 156 | M | 6 |
| 2 |  | 170 | M | 6 |
| 3 |  | 168 | M | 6 |
| 4 |  | 197 | M | 7 |
| 5 |  | 159 | M | 6 |
| 6 |  | 178 | M | 6 |
| 7 |  | 198 | M | 7 |
| 8 |  | 150 | M | 6 |
| 1 | RA group | 170 | M | 7 |
| 2 |  | 197 | M | 7 |
| 3 |  | 187 | M | 6 |
| 4 |  | 195 | M | 7 |
| 5 |  | 167 | M | 6 |
| 6 |  | 159 | M | 6 |
| 7 |  | 190 | M | 6 |
| 8 |  | 168 | M | 6 |
| 1 | Methotrexate group | 200 | M | 7 |
| 2 |  | 196 | M | 7 |
| 3 |  | 195 | M | 7 |
| 4 |  | 186 | M | 6 |
| 5 |  | 173 | M | 6 |
| 6 |  | 184 | M | 6 |
| 7 |  | 192 | M | 6 |
| 8 |  | 185 | M | 6 |
| 1 | Spirulina group | 198 | M | 7 |
| 2 |  | 173 | M | 6 |
| 3 |  | 163 | M | 6 |
| 4 |  | 191 | M | 6 |
| 5 |  | 199 | M | 7 |
| 6 |  | 150 | M | 6 |
| 7 |  | 187 | M | 6 |
| 8 |  | 179 | M | 6 |

**Table S2:** **Metabolites identiﬁed in *Spirulina* extract using UPLC-ESI-MS/MS in both negative and positive ionization modes**

| **No.** | **Rt (min.)** | **Identiﬁed compounds** | **Precursor ions** | **Molecular Formula** | **MS/MS Product Ions** | **Chemical class** |
| --- | --- | --- | --- | --- | --- | --- |
| 1 | 1.03 | 6-phosphogluconate | 277.2  [M+H]^+^ | C_6_H_13_O_10_P | 183-139-121 | Sugars |
| 2 | 1.42 | 3-Hydroxybutyrate | 105.2  [M+H]^+^ | C_4_H_8_O_3_ | 87-63 | Organic acids |
| 3 | 1.49 | Malonic acid | 103.34  [M−H]^−^ | C_3_H_4_O_4_ | 59 | Organic acids |
| 4 | 1.6 | 4-Hydroxybenzoic acid 4-O-glucoside | 299.2  [M−H]^−^ | C_13_H_16_O_8_ | 137-93 | Hydroxybenzoic acids |
| 5 | 1.69 | Malic acid | 133.12  [M−H]^−^ | C_4_H_5_O_5_ | 115 | Organic acids |
| 6 | 1.74 | 2-Hydroxyglutaric acid | 147.23  [M−H]^−^ | C_5_H_8_O_5_ | 103-59 | Organic acids |
| 7 | 2.01 | Isoferulic acid 3-sulfate | 273.15  [M−H]^−^ | C_10_H_10_O_7_S | 193-149 -178 | Phenolic acids |
| 8 | 2.07 | *p*-Hydroxybenzoic acid | 137.12  [M−H]^−^ | C_7_H_6_O_3_ | 93 | Hydroxybenzoic acids |
| 9 | 2.10 | Syringic acid | 197.15  [M−H]^−^ | C_9_H_9_O_5_ | 182-153-167 | Phenolic acids |
| 10 | 2.21 | Acetyl pyrrolidone | 128.14  [M+H]^+^ | C_6_H_9_NO_2_ | 85-43 | Nitrogenous compounds |
| 11 | 2.26 | Phloroglucinol | 125.15  [M−H]^−^ | C_6_H_5_O_3_ | 97 | Phenolic compounds |
| 12 | 2.35 | Caffeoylglycerol | 253.2  [M−H]^−^ | [C_12_H_14_O_6_](https://pubchem.ncbi.nlm.nih.gov/#query=C12H14O6) | 179 | Phenolic glycerides |
| 13 | 2.51 | Ellagic acid | 301.2  [M−H]^−^ | C_14_H_6_O_8_ | 257-229-185 | Hydrolysable tannins |
| 14 | 3.5 | Catechin | 289.2  [M−H]^−^ | C_15_H_14_O_6_ | 245- 205-139 | Flavonoids |
| 15 | 4.45 | Acetylornithine | 173.12  [M−H]^−^ | [C_7_H_14_N_2_O_3_](https://pubchem.ncbi.nlm.nih.gov/#query=C7H14N2O3) | 131-116-115-70 | Amino acids |
| 16 | 5.23 | Glutamyl cysteine | 251.35  [M+H]^+^ | C_8_H_14_N_2_O_5_S | 121-104-86 | Dipeptides |
| 17 | 5.44 | Phenyl pyruvic acid | 163.15  [M−H]^−^ | C_9_H_8_O_3_ | 144-116-88 | Organic acids |
| 18 | 5.94 | Brevifolin | 247.2  [M−H]^−^ | C_12_H_8_O_6_ | 219-191-163 | alkyl-phenylketones |
| 19 | 6.21 | 2-Isopropylmalate | 175.2  [M−H]^−^ | [C_7_H_12_O_5_](https://pubchem.ncbi.nlm.nih.gov/#query=C7H12O5) | 157-115-85 | Organic acids |
| 20 | 6.8 | Hydroxy ferulic acid | 209.2  [M−H]^−^ | C_10_H_10_O_5_ | 193-177-149 | Phenolic acids |
| 21 | 8.44 | Pantothenic acid | 218.2  [M−H]^−^ | [C_9_H_17_NO_5_](https://pubchem.ncbi.nlm.nih.gov/#query=C9H17NO5) | 200-182-174 | B vitamins |
| 22 | 8.75 | Rosmarinic acid | 359.42  [M−H]^−^ | C_18_H_16_O_8_ | 179-137-143 | Phenolic acids |
| 23 | 9.21 | 3,4,5,7-Tetrahydroxyisoﬂavanone | 289.3  [M+H]^+^ | C_15_H_12_O_6_ | 269, 259-136 | Flavonoids |
| 24 | 9.85 | Caffeic acid isoprenyl ester | 247.15  [M−H]^−^ | C_14_H_16_O_4_ | 179-135 | Phenolic acids |
| 25 | 10.5 | Phloroglucinol dimer derivative | 517.11  [M−H]^−^ | …….. | 247-125 | Phlorotannins |
| 26 | 11.25 | Glycyl tyrosine | 239.15  [M+H]^+^ | C_11_H_14_N_2_O_4_ | 165-119 | Dipeptides |
| 27 | 11.56 | Kaempferol -O-pentose | 417.42  [M−H]^−^ | C_20_H_19_O_11_ | 285-257-151 | Flavonoids |
| 28 | 11.67 | 3’-O-Methylequol | 273.25  [M+H]^+^ | C_16_H_16_O_4_ | 147- 123 | Flavonoids |
| 29 | 11.89 | Naringenin pentose | 405.35  [M+H]^+^ | C_20_H_21_O_10_ | 273-153-121 | Flavonoids |
| 30 | 12.14 | Kaempferol-methylether--O-glucoside | 463.35  [M+H]^+^ | C_22_H_22_O_11_ | 301-286-258-153 | Flavonoids |
| 31 | 12.25 | Hydroxydecanoic acid | 187.2  [M−H]^−^ | [C_10_H_20_O_3_](https://pubchem.ncbi.nlm.nih.gov/#query=C10H20O3) | 143-125 | Fatty acids |
| 32 | 12.55 | Taxifolin-O-rhamnoside | 449.35  [M−H]^−^ | C_21_H_22_O_11_ | 303-285-175-125 | Flavonoids |
| 33 | 12.63 | Rosmanol | 347.35  [M+H]^+^ | C_20_H_26_O_5_ | 301-231 | Terpenoids |
| 34 | 12.87 | Dimethoxy-luteolin-glucoside | 475.22  [M−H]^−^ | C_23_H_23_O_11_ | 315-285-241-179-151 | Flavonoids |
| 35 | 13.2 | Kaempferol-3-O-malonylglucoside | 533.15  [M−H]^−^ | C_24_H_22_O_14_ | 447-285-241-151 | Flavonoids |
| 36 | 13.53 | Cypellocarpin C | 521.5  [M+H]^+^ | [C_26_H_32_O_11_](https://pubchem.ncbi.nlm.nih.gov/#query=C26H32O11) | 337-189 | Phenol glycosides |
| 37 | 13.66 | 8-Pentadecenal | 225.25  [M+H]^+^ | [C_15_H_28_O](https://pubchem.ncbi.nlm.nih.gov/#query=C15H28O) | 207-197 | Fatty aldehydes |
| 38 | 13.72 | Quercetin | 301.15  [M−H]^−^ | C_15_H_10_O_7_ | 179-151 | Flavonoids |
| 39 | 14.68 | Lauramide | 200.12  [M+H]^+^ | C_12_H_25_NO | 156 | Fatty acid amides |
| 40 | 14.87 | Hydroxy myristamide | 244.21  [M+H]^+^ | C_14_H_29_NO_2_ | 226- 199-181 | Fatty acid amides |
| 41 | 15.33 | Oleanonic acid | 455.15  [M+H]^+^ | C_30_H_47_O_3_ | 437-409-395 | Terpenoids |
| 42 | 16.52 | Glycitein 7-O-glucuronide | 459.45  [M−H]^−^ | C_22_H_20_O_11_ | 283, 268, 224,133 | Flavonoids |
| 43 | 16.69 | Myristamide | 228.25  [M+H]^+^ | C_14_H_29_NO | 201 | Fatty acid amides |
| 44 | 17.4 | Taxifolin | 303.25  [M−H]^−^ | C_15_H_12_O_7_ | 285-175-125 | Flavonoids |
| 45 | 17.77 | Apigenin | 269.22  [M−H]^−^ | C_15_H_10_O_5_ | 255-165-151 | Flavonoids |
| 46 | 18.9 | Quercetin dimethylether | 329.35  [M−H]^−^ | C_17_H_14_O_7_ | 315-300-179-151 | Flavonoids |
| 47 | 20.04 | Fucophlorethol A | 373.12  [M−H]^−^ | C_18_H_14_O_9_ | 247-229 | Phlorotannins |
| 48 | 20.34 | Hydroxylauric acid | 215.25  [M−H]^−^ | [C_12_H_24_O_3_](https://pubchem.ncbi.nlm.nih.gov/#query=C12H24O3) | 171-153 | Fatty acids |
| 49 | 20.58 | Hydroxylinolenic acid | 293.3  [M−H]^−^ | C_18_H_29_O_3_ | 275-231 | Fatty acids |
| 50 | 20.97 | Pentadecenoic acid | 239.3  [M−H]^−^ | C_15_H_28_O_2_ | 195-193-155 | Fatty acids |
| 51 | 23.13 | Tetrafuhalol A | 529.22  [M−H]^−^ | C_24_H_18_O_14_ | 387 | Phlorotannins |
| 52 | 23.9 | Eckol derivative | 541.12  [M−H]^−^ | …… | 371-229 | Phlorotannins |
| 53 | 24.12 | Carnosic acid | 331.3  [M−H]^−^ | [C_20_H_28_O_4_](https://pubchem.ncbi.nlm.nih.gov/#query=C20H28O4) | 278-244 | Terpenoids |
| 54 | 24.3 | Stearidonic acid | 277.3  [M+H]^+^ | C_18_H_28_O_2_ | 255-233-162 | Fatty acids |
| 55 | 24.44 | Linolenic acid | 277.15  [M−H]^−^ | C_18_H_30_O_2_ | 233-111-69 | Fatty acids |
| 56 | 24.58 | Dihydroxypalmitic acid | 287.25  [M−H]^−^ | C_16_H_34_O_4_ | 269-251-207 | Fatty acids |
| 57 | 24.87 | Echinenone | 549.4  [M−H]^−^ | C_40_H_54_O | 531-203 | Carotenoids |
| 58 | 24.98 | Palmitoleic acid methyl ester | 267.3  [M−H]^−^ | C_17_H_32_O_2_ | 253-141 | Fatty acid esters |
| 59 | 25.7 | Hydroxylinoleic acid | 295.22  [M−H]^−^ | [C_18_H_32_O_3_](https://pubchem.ncbi.nlm.nih.gov/#query=C18H32O3) | 277-233-59 | Fatty acids |
| 60 | 25.9 | Eicosapentaenoic acid | 301.29  [M−H]^−^ | C_20_H_30_O_2_ | 257-108 | Fatty acids |
| 61 | 26.5 | Fucoxanthinol | 615.25  [M−H]^−^ | C_40_H_58_O_5_ | 597-579-147 | Carotenoids |
| 62 | 26.8 | Hydroxymyristic acid | 243.4  [M−H]^−^ | C_14_H_28_O_3_ | 225-181 | Fatty acids |
| 63 | 27.32 | Linoleic acid methyl ester | 293.35  [M−H]^−^ | [C_19_H_34_O_2_](https://pubchem.ncbi.nlm.nih.gov/#query=C19H34O2) | 277-233-111-59 | Fatty acid esters |
| 64 | 27.72 | Diadinoxanthin | 581.2  [M−H]^−^ | C_40_H_54_O_2_ | 563-545-221 | Carotenoids |
| 65 | 28.11 | Hydroxy palmitic acid | 271.35  [M−H]^−^ | C_16_H_31_O_3_ | 253-209 | Fatty acids |
| 66 | 28.3 | Campesterol | 401.6  [M+H]^+^ | C_28_H_48_O | 383- 268-147-161 | Phytosterols |
| 67 | 28.45 | Hydroxystearic acid | 299.3  [M−H]^−^ | C_18_H_36_O_3_ | 281-237 | Fatty acids |
| 68 | 28.6 | Oleic acid | 281.2  [M−H]^−^ | C_18_H_34_O_2_ | 237-124-59 | Fatty acids |
| 69 | 28.75 | Nonadecenoic acid | 295.4  [M−H]^−^ | C_19_H_36_O_2_ | 251-128 | Fatty acids |
| 70 | 29.42 | Nonadecanoic acid | 297.23  [M−H]^−^ | C_19_H_38_O2 | 253 | Fatty acids |
| 71 | 29.8 | Campestanol | 425.6  [M + Na]^+^ | C_28_H_50_O | 385-149-163 | Phytosterols |
| 72 | 30.03 | Betulinic acid | 455.12  [M−H]^−^ | C_30_H_48_O_3_ | 440- 435-407 | Terpenoids |

**Table S3: Effect of different *S. Platensis* doses on serum oxidative stress and inflammatory markers of RA rats**

| Serum markers | Normal group (NG) | RA model  (MG) | Low dose  (200 mg/kg/day) | Medium dose  (400 mg/kg/day) | High dose  (800 mg/kg/day) |
| --- | --- | --- | --- | --- | --- |
| MDA (nmol/mL) | **0.30 ± 0.02** | **1.9^a^ ± 0.03** | **1.6^a^ ± 0.02** | **1.2^ab^ ± 0.03** | **0.83^ab^± 0.05** |
| SOD (U/mL) | **2.5 ± 0.19** | **0.93^a^ ± 0.12** | **0.98^a^ ± 0.14** | **1.4^ab^ ± 0.02** | **1.9^ab^± 0.07** |
| RF (IU/mL) | **5 ± 0.73** | **15.8^a^ ± 0.62** | **12.6^a^ ± 0.63** | **9.8^ab^ ± 0.79** | **8.7^ab^ ± 0.3** |
| MCP-1 (pg/mL) | **27.7 ± 5** | **162.5^a^ ± 5.2** | **125.2^a^ ± 6** | **85.6^ab^ ± 4** | **68.3^ab^ ± 5** |

NG: normal group, MG: RA model group, *Spirulina* extract-treated groups, MDA; Malondialdehyde, SOD; Superoxide dismutase, RF; Rheumatoid factor, MCP-1; Monocyte Chemoattractant Protein-1. Values are means ± SD (n=3) (one-way ANOVA followed by Tukey post hoc test) at *p* ≤ 0.05.

**Table S4: All endogenous metabolites detected in NG, MG and SP and the content of each variable (peak areas)**

| Polarity | Metabolites | NG | NG' | NG'' | NG''' | NG'''' | MG | MG' | MG'' | MG''' | MG'''' | SG | SG' | SG'' | SG''' | SG'''' |
| --- | --- | --- | --- | --- | --- | --- | --- | --- | --- | --- | --- | --- | --- | --- | --- | --- |
| N | Citric acid | 12134111.72 | 10920700.54 | 13347522.89 | 9828630.489 | 14318251.82 | 0 | 0 | 0 | 0 | 0 | 9693409.92 | 8724068.928 | 10856619.11 | 7270057.44 | 11825960.1 |
| P | 104.2 | 36096004 | 32486403.6 | 39705604.4 | 29237763.24 | 42593284.72 | 58770193 | 52893173.7 | 64647212.3 | 68761125.81 | 48779260.19 | 40096004 | 36086403.6 | 44907524.48 | 30072003 | 48917124.88 |
| P | 122.4 | 9165640 | 8249076 | 10082204 | 7424168.4 | 10815455.2 | 4967650 | 4470885 | 5464415 | 5812150.5 | 4123149.5 | 8154640 | 7339176 | 9133196.8 | 6115980 | 9948660.8 |
| P | 100.1 | 150979612.4 | 135881651.1 | 166077573.6 | 122293486 | 178155942.6 | 99770315.73 | 89793284.16 | 109747347.3 | 116731269.4 | 82809362.06 | 119872456.4 | 107885210.7 | 134257151.1 | 89904342.28 | 146244396.8 |
| P | 101.1 | 28653210 | 25787889 | 31518531 | 23209100.1 | 33810787.8 | 3765120 | 3388608 | 4141632 | 4405190.4 | 3125049.6 | 8976590 | 8078931 | 10053780.8 | 6732442.5 | 10951439.8 |
| P | 102.2 | 2134502 | 1921051.8 | 2347952.2 | 1728946.62 | 2518712.36 | 834520 | 751068 | 917972 | 976388.4 | 692651.6 | 543212 | 488890.8 | 608397.44 | 407409 | 662718.64 |
| P | 152.2 | 55643230 | 50078907 | 61207553 | 45071016.3 | 65659011.4 | 20554320 | 18498888 | 22609752 | 24048554.4 | 17060085.6 | 34523167 | 31070850.3 | 38665947.04 | 25892375.25 | 42118263.74 |
| P | 114.3 | 759087 | 683178.3 | 834995.7 | 614860.47 | 895722.66 | 3324210 | 2991789 | 3656631 | 3889325.7 | 2759094.3 | 578603 | 520742.7 | 648035.36 | 433952.25 | 705895.66 |
| P | 110.1 | 36212654 | 32591388.6 | 39833919.4 | 29332249.74 | 42730931.72 | 5343207 | 4808886.3 | 5877527.7 | 6251552.19 | 4434861.81 | 53232107 | 47908896.3 | 59619959.84 | 39924080.25 | 64943170.54 |
| P | 110.2 | 69563230 | 62606907 | 76519553 | 56346216.3 | 82084611.4 | 5290870 | 4761783 | 5819957 | 6190317.9 | 4391422.1 | 55783450 | 50205105 | 62477464 | 41837587.5 | 68055809 |
| P | 110.3 | 3154890 | 2839401 | 3470379 | 2555460.9 | 3722770.2 | 2654301 | 2388870.9 | 2919731.1 | 3105532.17 | 2203069.83 | 5654300 | 5088870 | 6332816 | 4240725 | 6898246 |
| N | 127.2 | 4654328 | 4188895.2 | 5119760.8 | 3770005.68 | 5492107.04 | 2865641 | 2579076.9 | 3152205.1 | 3352799.97 | 2378482.03 | 1444122 | 1299709.8 | 1617416.64 | 1083091.5 | 1761828.84 |
| N | *Fumerate* | 3.75E+06 | 3378710.7 | 4129535.3 | 3040839.63 | 4429865.14 | 0 | 0 | 0 | 0 | 0 | 3865231 | 3478707.9 | 4329058.72 | 2898923.25 | 4715581.82 |
| N | Succinic acid | 1.74E+06 | 1569188.7 | 1917897.3 | 1412269.83 | 2057380.74 | 49787654 | 44808888.6 | 54766419.4 | 58251555.18 | 41323752.82 | 25221220 | 22699098 | 28247766.4 | 18915915 | 30769888.4 |
| P | 118.1 | 3678912 | 3311020.8 | 4046803.2 | 2979918.72 | 4341116.16 | 3213453 | 2892107.7 | 3534798.3 | 3759740.01 | 2667165.99 | 1786543 | 1607888.7 | 2000928.16 | 1339907.25 | 2179582.46 |
| P | 124.3 | 3108200 | 2797380 | 3419020 | 2517642 | 3667676 | 17435211 | 15691689.9 | 19178732.1 | 20399196.87 | 14471225.13 | 1498200 | 1348380 | 1677984 | 1123650 | 1827804 |
| P | 221.1 | 2223153 | 2000837.7 | 2445468.3 | 1800753.93 | 2623320.54 | 1876543 | 1688888.7 | 2064197.3 | 2195555.31 | 1557530.69 | 0 | 0 | 0 | 0 | 0 |
| N | *215.2* | 20654320 | 18588888 | 22719752 | 16729999.2 | 24372097.6 | 25789430 | 23210487 | 28368373 | 30173633.1 | 21405226.9 | 8644430 | 7779987 | 9681761.6 | 6483322.5 | 10546204.6 |
| N | *266.9* | 2765430 | 2488887 | 3041973 | 2239998.3 | 3263207.4 | 2876900 | 2589210 | 3164590 | 3365973 | 2387827 | 1498760 | 1348884 | 1678611.2 | 1124070 | 1828487.2 |
| N | 269.2 | 228654321 | 205788888.9 | 251519753.1 | 185210000 | 269812098.8 | 19341232 | 17407108.8 | 21275355.2 | 22629241.44 | 16053222.56 | 7554323 | 6798890.7 | 8460841.76 | 5665742.25 | 9216274.06 |
| N | 140.3 | 4432123 | 3988910.7 | 4875335.3 | 3590019.63 | 5229905.14 | 2316754 | 2085078.6 | 2548429.4 | 2710602.18 | 1922905.82 | 0 | 0 | 0 | 0 | 0 |
| N | 141.3 | 3124210 | 2811789 | 3436631 | 2530610.1 | 3686567.8 | 2134213 | 1920791.7 | 2347634.3 | 2497029.21 | 1771396.79 | 0 | 0 | 0 | 0 | 0 |
| N | 144.4 | 21245460 | 19120914 | 23370006 | 17208822.6 | 25069642.8 | 8403210 | 7562889 | 9243531 | 9831755.7 | 6974664.3 | 17232130 | 15508917 | 19299985.6 | 12924097.5 | 21023198.6 |
| N | 144.2 | 1931220 | 1738098 | 2124342 | 1564288.2 | 2278839.6 | 2545321 | 2290788.9 | 2799853.1 | 2978025.57 | 2112616.43 | 1644342 | 1479907.8 | 1841663.04 | 1233256.5 | 2006097.24 |
| N | capric acid | 3.77E+07 | 33930000 | 41470000 | 30537000 | 44486000 | 1765405 | 1588864.5 | 1941945.5 | 2065523.85 | 1465286.15 | 2.08E+07 | 18720000 | 23296000 | 15600000 | 25376000 |
| N | 145.4 | 3.79E+07 | 34078887 | 41651973 | 30670998.3 | 44681207.4 | 4653432 | 4188088.8 | 5118775.2 | 5444515.44 | 3862348.56 | 27876760 | 25089084 | 31221971.2 | 20907570 | 34009647.2 |
| N | 149.1 | 1876123 | 1688510.7 | 2063735.3 | 1519659.63 | 2213825.14 | 751230 | 676107 | 826353 | 878939.1 | 623520.9 | 0 | 0 | 0 | 0 | 0 |
| N | *glutamic acid* | 5.68E+06 | 5111017.2 | 6246798.8 | 4599915.48 | 6701111.44 | 0 | 0 | 0 | 0 | 0 | 782341 | 704106.9 | 876221.92 | 586755.75 | 954456.02 |
| N | 151.1 | 2765121 | 2488608.9 | 3041633.1 | 2239748.01 | 3262842.78 | 2512345 | 2261110.5 | 2763579.5 | 2939443.65 | 2085246.35 | 1876234 | 1688610.6 | 2101382.08 | 1407175.5 | 2289005.48 |
| P | L-Leucine | 1.81E+07 | 16295559.74 | 19916795.23 | 14666003.76 | 21365289.43 | 9269264.745 | 8342338.271 | 10196191.22 | 10845039.75 | 7693489.738 | 3417666.315 | 3075899.684 | 3827786.273 | 2563249.736 | 4169552.904 |
| N | 117.2 | 3554378 | 3198940.2 | 3909815.8 | 2879046.18 | 4194166.04 | 15345120 | 13810608 | 16879632 | 17953790.4 | 12736449.6 | 7522321 | 6770088.9 | 8424999.52 | 5641740.75 | 9177231.62 |
| P | 257.2 | 1876510 | 1688859 | 2064161 | 1519973.1 | 2214281.8 | 954320 | 858888 | 1049752 | 1116554.4 | 792085.6 | 487650 | 438885 | 546168 | 365737.5 | 594933 |
| P | 130.2 | 68275984 | 61448385.6 | 75103582.4 | 55303547.04 | 80565661.12 | 39147121 | 35232408.9 | 43061833.1 | 45802131.57 | 32492110.43 | 60956408 | 54860767.2 | 68271176.96 | 45717306 | 74366817.76 |
| P | Histidine | 234123 | 210710.7 | 257535.3 | 189639.63 | 276265.14 | 108765 | 97888.5 | 119641.5 | 127255.05 | 90274.95 | 129552 | 116596.8 | 145098.24 | 97164 | 158053.44 |
| N | 157.3 | 2513672 | 2262304.8 | 2765039.2 | 2036074.32 | 2966132.96 | 3657891 | 3292101.9 | 4023680.1 | 4279732.47 | 3036049.53 | 1855551 | 1669995.9 | 2078217.12 | 1391663.25 | 2263772.22 |
| N | 191.3 | 12987601 | 11688840.9 | 14286361.1 | 10519956.81 | 15325369.18 | 13245408 | 11920867.2 | 14569948.8 | 15497127.36 | 10993688.64 | 7987678 | 7188910.2 | 8946199.36 | 5990758.5 | 9744967.16 |
| N | *gluconic acid* | 1.52E+06 | 1369177.2 | 1673438.8 | 1232259.48 | 1795143.44 | 8234310 | 7410879 | 9057741 | 9634142.7 | 6834477.3 | 2787823 | 2509040.7 | 3122361.76 | 2090867.25 | 3401144.06 |
| P | 261.2 | 1576531 | 1418877.9 | 1734184.1 | 1276990.11 | 1860306.58 | 98765 | 88888.5 | 108641.5 | 115555.05 | 81974.95 | 0 | 0 | 0 | 0 | 0 |
| N | 307.2 | 776531 | 698877.9 | 854184.1 | 628990.11 | 916306.58 | 597653 | 537887.7 | 657418.3 | 699254.01 | 496051.99 | 676531 | 608877.9 | 757714.72 | 507398.25 | 825367.82 |
| N | 349.2 | 1076531 | 968877.9 | 1184184.1 | 871990.11 | 1270306.58 | 1376531 | 1238877.9 | 1514184.1 | 1610541.27 | 1142520.73 | 1176531 | 1058877.9 | 1317714.72 | 882398.25 | 1435367.82 |
| P | 110.1 | 6.76E+07 | 60877933.29 | 74406362.91 | 54790139.96 | 79817734.76 | 3.26E+07 | 29340541.85 | 35860662.26 | 38142704.4 | 27058499.7 | 5.11E+07 | 45998959.85 | 57243150.03 | 38332466.54 | 62354145.57 |
| P | Methionine | 5069152.665 | 4562237.398 | 5576067.931 | 4106013.659 | 5981600.145 | 11548070.41 | 10393263.37 | 12702877.45 | 13511242.38 | 9584898.44 | 3.63E+06 | 3270381.021 | 4069807.493 | 2725317.517 | 4433183.162 |
| N | 129.3 | 1634512 | 1471060.8 | 1797963.2 | 1323954.72 | 1928724.16 | 0 | 0 | 0 | 0 | 0 | 0 | 0 | 0 | 0 | 0 |
| N | 135.2 | 341267 | 307140.3 | 375393.7 | 276426.27 | 402695.06 | 187654 | 168888.6 | 206419.4 | 219555.18 | 155752.82 | 58764 | 52887.6 | 65815.68 | 44073 | 71692.08 |
| N | 125.4 | 11176520 | 10058868 | 12294172 | 9052981.2 | 13188293.6 | 8305660 | 7475094 | 9136226 | 9717622.2 | 6893697.8 | 4654340 | 4188906 | 5212860.8 | 3490755 | 5678294.8 |
| P | 122.3 | 2.34E+07 | 21045887.01 | 25722750.78 | 18941298.3 | 27593496.3 | 9024205.545 | 8121784.991 | 9926626.1 | 10558320.49 | 7490090.602 | 1.32E+07 | 11850561.82 | 14747365.82 | 9875468.182 | 16064094.91 |
| P | 205.2 | 17232509.88 | 15509258.89 | 18955760.87 | 13958333 | 20334361.66 | 9601930.8 | 8641737.72 | 10562123.88 | 11234259.04 | 7969602.564 | 16514846.68 | 14863362.01 | 18496628.28 | 12386135.01 | 20148112.94 |
| P | 166.2 | 7120529.52 | 6408476.568 | 7832582.472 | 5767628.911 | 8402224.834 | 4051188.165 | 3646069.349 | 4456306.982 | 4739890.153 | 3362486.177 | 6530920.52 | 5877828.468 | 7314630.982 | 4898190.39 | 7967723.034 |
| N | 225.2 | 7065431 | 6358887.9 | 7771974.1 | 5722999.11 | 8337208.58 | 5432120 | 4888908 | 5975332 | 6355580.4 | 4508659.6 | 2564320 | 2307888 | 2872038.4 | 1923240 | 3128470.4 |
| N | 226.2 | 3124530 | 2812077 | 3436983 | 2530869.3 | 3686945.4 | 0 | 0 | 0 | 0 | 0 | 0 | 0 | 0 | 0 | 0 |
| N | 228.2 | 145432 | 130888.8 | 159975.2 | 117799.92 | 171609.76 | 112345 | 101110.5 | 123579.5 | 131443.65 | 93246.35 | 83452 | 75106.8 | 93466.24 | 62589 | 101811.44 |
| P | Tryptophan | 17658902 | 15893011.8 | 19424792.2 | 14303710.62 | 20837504.36 | 6987650 | 6288885 | 7686415 | 8175550.5 | 5799749.5 | 1.44E+06 | 1296000 | 1612800 | 1080000 | 1756800 |
| P | 157.2 | 143211 | 128889.9 | 157532.1 | 116000.91 | 168988.98 | 176542 | 158887.8 | 194196.2 | 206554.14 | 146529.86 | 187651 | 168885.9 | 210169.12 | 140738.25 | 228934.22 |
| P | 162.2 | 1713121 | 1541808.9 | 1884433.1 | 1387628.01 | 2021482.78 | 2645320 | 2380788 | 2909852 | 3095024.4 | 2195615.6 | 2843120 | 2558808 | 3184294.4 | 2132340 | 3468606.4 |
| N | 165.2 | 2665430 | 2398887 | 2931973 | 2158998.3 | 3145207.4 | 0 | 0 | 0 | 0 | 0 | 87652 | 78886.8 | 98170.24 | 65739 | 106935.44 |
| N | 171.3 | 162312 | 146080.8 | 178543.2 | 131472.72 | 191528.16 | 137512 | 123760.8 | 151263.2 | 160889.04 | 114134.96 | 0 | 0 | 0 | 0 | 0 |
| N | 185.3 | 23123 | 20810.7 | 25435.3 | 18729.63 | 27285.14 | 43123 | 38810.7 | 47435.3 | 50453.91 | 35792.09 | 47512 | 42760.8 | 53213.44 | 35634 | 57964.64 |
| N | 187.6 | 565412 | 508870.8 | 621953.2 | 457983.72 | 667186.16 | 267541 | 240786.9 | 294295.1 | 313022.97 | 222059.03 | 204312 | 183880.8 | 228829.44 | 153234 | 249260.64 |
| N | 163.3 | 3455454 | 3109908.6 | 3800999.4 | 2798917.74 | 4077435.72 | 1345122 | 1210609.8 | 1479634.2 | 1573792.74 | 1116451.26 | 2345143 | 2110628.7 | 2626560.16 | 1758857.25 | 2861074.46 |
| P | Methyllysine | 2.18E+07 | 19584305.46 | 23936373.33 | 17625874.91 | 25677200.49 | 1.11E+07 | 9973068.781 | 12189306.29 | 12964989.42 | 9197385.654 | 1.85E+07 | 16637901.23 | 20704943.75 | 13864917.69 | 22553599.44 |
| N | 187.2 | 2765432 | 2488888.8 | 3041975.2 | 2239999.92 | 3263209.76 | 1678341 | 1510506.9 | 1846175.1 | 1963658.97 | 1393023.03 | 2687123 | 2418410.7 | 3009577.76 | 2015342.25 | 3278290.06 |
| N | 161.2 | 198734 | 178860.6 | 218607.4 | 160974.54 | 234506.12 | 145612 | 131050.8 | 160173.2 | 170366.04 | 120857.96 | 232122 | 208909.8 | 259976.64 | 174091.5 | 283188.84 |
| N | Myristic acid | 12345125 | 11110612.5 | 13579637.5 | 9999551.25 | 14567247.5 | 5123121 | 4610808.9 | 5635433.1 | 5994051.57 | 4252190.43 | 6789564 | 6110607.6 | 7604311.68 | 5092173 | 8283268.08 |
| P | 200.11 | 17616111 | 15854499.9 | 19377722.1 | 14269049.91 | 20787010.98 | 19876131 | 17888517.9 | 21863744.1 | 23255073.27 | 16497188.73 | 18676131 | 16808517.9 | 20917266.72 | 14007098.25 | 22784879.82 |
| P | 514.2 | 345123 | 310610.7 | 379635.3 | 279549.63 | 407245.14 | 2634511 | 2371059.9 | 2897962.1 | 3082377.87 | 2186644.13 | 375123 | 337610.7 | 420137.76 | 281342.25 | 457650.06 |
| N | 179.2 | 93124 | 83811.6 | 102436.4 | 75430.44 | 109886.32 | 176812 | 159130.8 | 194493.2 | 206870.04 | 146753.96 | 0 | 0 | 0 | 0 | 0 |
| N | 181.2 | 1675340 | 1507806 | 1842874 | 1357025.4 | 1976901.2 | 1876520 | 1688868 | 2064172 | 2195528.4 | 1557511.6 | 3543320 | 3188988 | 3968518.4 | 2657490 | 4322850.4 |
| N | Sphingosine-1-phosphate | 0 | 0 | 0 | 0 | 0 | 5266550.245 | 4739895.221 | 5793205.27 | 6161863.787 | 4371236.703 | 0 | 0 | 0 | 0 | 0 |
| N | 386.2 | 135643 | 122078.7 | 149207.3 | 109870.83 | 160058.74 | 113243 | 101918.7 | 124567.3 | 132494.31 | 93991.69 | 0 | 0 | 0 | 0 | 0 |
| N | 833.2 | 2501527.875 | 2251375.087 | 2751680.662 | 2026237.579 | 2951802.892 | 1388208.535 | 1249387.682 | 1527029.389 | 1624203.986 | 1152213.084 | 0 | 0 | 0 | 0 | 0 |
| P | 152.4 | 4321675 | 3889507.5 | 4753842.5 | 3500556.75 | 5099576.5 | 11232331 | 10109097.9 | 12355564.1 | 13141827.27 | 9322834.73 | 3432321 | 3089088.9 | 3844199.52 | 2574240.75 | 4187431.62 |
| N | 217.5 | 6231411 | 5608269.9 | 6854552.1 | 5047442.91 | 7353064.98 | 0 | 0 | 0 | 0 | 0 | 0 | 0 | 0 | 0 | 0 |
| N | 221.3 | 3935120 | 3541608 | 4328632 | 3187447.2 | 4643441.6 | 0 | 0 | 0 | 0 | 0 | 0 | 0 | 0 | 0 | 0 |
| N | Cholesterol | 3398967.285 | 3059070.556 | 3738864.013 | 2753163.501 | 4010781.396 | 1218626.82 | 1096764.138 | 1340489.502 | 1425793.379 | 1011460.261 | 1306143.225 | 1175528.902 | 1462880.412 | 979607.4187 | 1593494.734 |
| N | 194.2 | 1543120 | 1388808 | 1697432 | 1249927.2 | 1820881.6 | 2813560 | 2532204 | 3094916 | 3291865.2 | 2335254.8 | 2056430 | 1850787 | 2303201.6 | 1542322.5 | 2508844.6 |
| P | 430.2 | 1513451 | 1362105.9 | 1664796.1 | 1225895.31 | 1785872.18 | 28134513 | 25321061.7 | 30947964.3 | 32917380.21 | 23351645.79 | 2012221 | 1810998.9 | 2253687.52 | 1509165.75 | 2454909.62 |
| N | 201.2 | 1876543 | 1688888.7 | 2064197.3 | 1519999.83 | 2214320.74 | 987654 | 888888.6 | 1086419.4 | 1155555.18 | 819752.82 | 566654 | 509988.6 | 634652.48 | 424990.5 | 691317.88 |
| P | 518.2 | 1201527.875 | 1081375.087 | 1321680.662 | 973237.5787 | 1417802.892 | 2588208.535 | 2329387.682 | 2847029.389 | 3028203.986 | 2148213.084 | 1456955.535 | 1311259.982 | 1631790.199 | 1092716.651 | 1777485.753 |
| N | 208.2 | 1886534 | 1697880.6 | 2075187.4 | 1528092.54 | 2226110.12 | 1687645 | 1518880.5 | 1856409.5 | 1974544.65 | 1400745.35 | 1504321 | 1353888.9 | 1684839.52 | 1128240.75 | 1835271.62 |
| N | 210.3 | 1787540 | 1608786 | 1966294 | 1447907.4 | 2109297.2 | 2478610 | 2230749 | 2726471 | 2899973.7 | 2057246.3 | 1475340 | 1327806 | 1652380.8 | 1106505 | 1799914.8 |
| P | 520.2 | 1.95E+07 | 17567009.62 | 21470789.54 | 15810308.66 | 23032301.5 | 2.48E+07 | 22324622.75 | 27285650.03 | 29022009.58 | 20588263.2 | 1.08E+07 | 9719671.072 | 12095590.67 | 8099725.894 | 13175554.12 |
| N | 249.2 | 87765 | 78988.5 | 96541.5 | 71089.65 | 103562.7 | 167654 | 150888.6 | 184419.4 | 196155.18 | 139152.82 | 147654 | 132888.6 | 165372.48 | 110740.5 | 180137.88 |
| P | 496.3 | 3.32E+07 | 29848494.99 | 36481493.88 | 26863645.49 | 39134693.43 | 2.79E+07 | 25121768.42 | 30704383.63 | 32658298.95 | 23167853.1 | 2.06E+07 | 18554566.1 | 23090126.71 | 15462138.42 | 25151745.16 |
| P | 544.2 | 7666297.785 | 6899668.006 | 8432927.563 | 6209701.206 | 9046231.386 | 1.13E+07 | 10174948.43 | 12436048.09 | 13227432.96 | 9383563.556 | 6205944.345 | 5585349.91 | 6950657.666 | 4654458.259 | 7571252.101 |
| P | 522.3 | 1.09E+07 | 9799523.95 | 11977195.94 | 8819571.555 | 12848264.74 | 1.51E+07 | 13617494.33 | 16643604.19 | 17702742.63 | 12558355.89 | 5205944.345 | 4685349.91 | 5830657.666 | 3904458.259 | 6351252.101 |
| P | 546.2 | 1197747.945 | 1077973.151 | 1317522.74 | 970175.8355 | 1413342.575 | 1555361.44 | 1399825.296 | 1710897.584 | 1819772.885 | 1290949.995 | 1213293.285 | 1091963.957 | 1358888.479 | 909969.9638 | 1480217.808 |
| P | 279.2 | 2435671 | 2192103.9 | 2679238.1 | 1972893.51 | 2874091.78 | 3152456 | 2837210.4 | 3467701.6 | 3688373.52 | 2616538.48 | 1856123 | 1670510.7 | 2078857.76 | 1392092.25 | 2264470.06 |
| P | 524.3 | 3092656.675 | 2783391.007 | 3401922.342 | 2505051.907 | 3649334.876 | 2.36E+07 | 21244577.62 | 25965594.86 | 27617950.9 | 19592221.58 | 1.49E+07 | 13378442.31 | 16648728.21 | 11148701.93 | 18135221.8 |
| N | 636.3 | 267543 | 240788.7 | 294297.3 | 216709.83 | 315700.74 | 95432 | 85888.8 | 104975.2 | 111655.44 | 79208.56 | 187654 | 168888.6 | 210172.48 | 140740.5 | 228937.88 |
| N | 638.2 | 178654 | 160788.6 | 196519.4 | 144709.74 | 210811.72 | 213456 | 192110.4 | 234801.6 | 249743.52 | 177168.48 | 176543 | 158888.7 | 197728.16 | 132407.25 | 215382.46 |
| N | 640.3 | 453212 | 407890.8 | 498533.2 | 367101.72 | 534790.16 | 376542 | 338887.8 | 414196.2 | 440554.14 | 312529.86 | 254623 | 229160.7 | 285177.76 | 190967.25 | 310640.06 |
| N | 656.2 | 0 | 0 | 0 | 0 | 0 | 56432 | 50788.8 | 62075.2 | 66025.44 | 46838.56 | 0 | 0 | 0 | 0 | 0 |
| N | 666.2 | 254621 | 229158.9 | 280083.1 | 206243.01 | 300452.78 | 0 | 0 | 0 | 0 | 0 | 0 | 0 | 0 | 0 | 0 |
| N | (18:1/18:1) Phosphatidic acid | 7.27E+07 | 65448608.35 | 79992743.54 | 58903747.52 | 85810397.62 | 1.27E+07 | 11448608.35 | 13992743.54 | 14883190.86 | 10558161.03 | 2704452.915 | 2434007.624 | 3028987.265 | 2028339.686 | 3299432.556 |
| N | (20:2/18:2) Phosphatidylcholine | 0.00E+00 | 0 | 0 | 0 | 0 | 5.72E+07 | 51477337.26 | 62916745.53 | 66920538.43 | 47473544.36 | 2.56E+07 | 23073681.03 | 28713914.17 | 19228067.53 | 31277656.51 |
| P | Arachidonic acid | 1238273.94 | 1114446.546 | 1362101.334 | 1003001.891 | 1461163.249 | 3073004.58 | 2765704.122 | 3380305.038 | 3595415.359 | 2550593.801 | 1274590.245 | 1147131.22 | 1427541.074 | 955942.6837 | 1555000.099 |
| N | 700.1 | 267545 | 240790.5 | 294299.5 | 216711.45 | 315703.1 | 334567 | 301110.3 | 368023.7 | 391443.39 | 277690.61 | 278980 | 251082 | 312457.6 | 209235 | 340355.6 |
| N | 273.3 | 178654 | 160788.6 | 196519.4 | 144709.74 | 210811.72 | 123412 | 111070.8 | 135753.2 | 144392.04 | 102431.96 | 0 | 0 | 0 | 0 | 0 |
| P | 281.2 | 3203323.935 | 2882991.541 | 3523656.328 | 2594692.387 | 3779922.243 | 2037590.365 | 1833831.328 | 2241349.401 | 2383980.727 | 1691200.003 | 2535428.95 | 2281886.055 | 2839680.424 | 1901571.712 | 3093223.319 |
| N | (18:1/18:0) Phosphatidic acid | 1.25E+07 | 11206474.61 | 13696802.3 | 10085827.14 | 14692933.37 | 3773874.62 | 3396487.158 | 4151262.082 | 4415433.305 | 3132315.935 | 8757711.705 | 7881940.534 | 9808637.11 | 6568283.779 | 10684408.28 |
| P | 5,6-Epoxy-8,11,14-eicosatrienoic acid | 0 | 0 | 0 | 0 | 0 | 2042898.675 | 1838608.807 | 2247188.542 | 2390191.45 | 1695605.9 | 0 | 0 | 0 | 0 | 0 |
| N | 277.3 | 145378 | 130840.2 | 159915.8 | 117756.18 | 171546.04 | 0 | 0 | 0 | 0 | 0 | 453902 | 408511.8 | 508370.24 | 340426.5 | 553760.44 |
| P | 325.3 | 2612344 | 2351109.6 | 2873578.4 | 2115998.64 | 3082565.92 | 21344120 | 19209708 | 23478532 | 24972620.4 | 17715619.6 | 5324120 | 4791708 | 5963014.4 | 3993090 | 6495426.4 |
| P | 397.1 | 398761 | 358884.9 | 438637.1 | 322996.41 | 470537.98 | 0 | 0 | 0 | 0 | 0 | 0 | 0 | 0 | 0 | 0 |
| P | 349.2 | 134250 | 120825 | 147675 | 108742.5 | 158415 | 0 | 0 | 0 | 0 | 0 | 0 | 0 | 0 | 0 | 0 |
| P | (16:0/18:2) Phosphatidylcholine | 0 | 0 | 0 | 0 | 0 | 17999866.59 | 16199879.93 | 19799853.24 | 21059843.9 | 14939889.27 | 2626004.585 | 2363404.127 | 2941125.135 | 1969503.439 | 3203725.594 |
| N | 307.3 | 76543 | 68888.7 | 84197.3 | 61999.83 | 90320.74 | 345546 | 310991.4 | 380100.6 | 404288.82 | 286803.18 | 67543 | 60788.7 | 75648.16 | 50657.25 | 82402.46 |
| N | 309.1 | 1786543 | 1607888.7 | 1965197.3 | 1447099.83 | 2108120.74 | 0 | 0 | 0 | 0 | 0 | 0 | 0 | 0 | 0 | 0 |
| N | 311.3 | 1745671 | 1571103.9 | 1920238.1 | 1413993.51 | 2059891.78 | 94512 | 85060.8 | 103963.2 | 110579.04 | 78444.96 | 0 | 0 | 0 | 0 | 0 |
| N | 313.2 | 798654 | 718788.6 | 878519.4 | 646909.74 | 942411.72 | 0 | 0 | 0 | 0 | 0 | 0 | 0 | 0 | 0 | 0 |
| N | 315.2 | 1565432 | 1408888.8 | 1721975.2 | 1267999.92 | 1847209.76 | 1987650 | 1788885 | 2186415 | 2325550.5 | 1649749.5 | 0 | 0 | 0 | 0 | 0 |
| N | 804.2 | 0 | 0 | 0 | 0 | 0 | 678450 | 610605 | 746295 | 793786.5 | 563113.5 | 0 | 0 | 0 | 0 | 0 |
| N | 812.1 | 112345 | 101110.5 | 123579.5 | 90999.45 | 132567.1 | 132450 | 119205 | 145695 | 154966.5 | 109933.5 | 78765 | 70888.5 | 88216.8 | 59073.75 | 96093.3 |
| N | (20:0/18:2) Phosphatidylserine | 665342 | 598807.8 | 731876.2 | 538927.02 | 785103.56 | 0 | 0 | 0 | 0 | 0 | 0 | 0 | 0 | 0 | 0 |
| N | 826.2 | 0 | 0 | 0 | 0 | 0 | 690769 | 621692.1 | 759845.9 | 808199.73 | 573338.27 | 0 | 0 | 0 | 0 | 0 |
| N | 841.1 | 76545 | 68890.5 | 84199.5 | 62001.45 | 90323.1 | 0 | 0 | 0 | 0 | 0 | 0 | 0 | 0 | 0 | 0 |
| N | 833 | 234244 | 210819.6 | 257668.4 | 189737.64 | 276407.92 | 123333 | 110999.7 | 135666.3 | 144299.61 | 102366.39 | 234244 | 210819.6 | 262353.28 | 175683 | 285777.68 |
| N | 855.3 | 1245671 | 1121103.9 | 1370238.1 | 1008993.51 | 1469891.78 | 564443 | 507998.7 | 620887.3 | 660398.31 | 468487.69 | 209753 | 188777.7 | 234923.36 | 157314.75 | 255898.66 |
| N | 905 | 298765 | 268888.5 | 328641.5 | 241999.65 | 352542.7 | 164543 | 148088.7 | 180997.3 | 192515.31 | 136570.69 | 0 | 0 | 0 | 0 | 0 |
| P | (20:4/16:0) Phosphatidylcholine | 0 | 0 | 0 | 0 | 0 | 1.53E+07 | 13741998.85 | 16795776.38 | 17864598.51 | 12673176.72 | 0 | 0 | 0 | 0 | 0 |
| N | 530 | 32156 | 28940.4 | 35371.6 | 26046.36 | 37944.08 | 167541 | 150786.9 | 184295.1 | 196022.97 | 139059.03 | 76543 | 68888.7 | 85728.16 | 57407.25 | 93382.46 |
| N | 538.2 | 476549 | 428894.1 | 524203.9 | 386004.69 | 562327.82 | 987654 | 888888.6 | 1086419.4 | 1155555.18 | 819752.82 | 387651 | 348885.9 | 434169.12 | 290738.25 | 472934.22 |
|  | (20:4/18:0) Phosphatidylcholine | 0 | 0 | 0 | 0 | 0 | 17058902 | 16547890 | 18768902 | 15987909 | 17989904 | 1675678 | 1789670 | 1876789 | 1546760 | 1498760 |
| N | 554.2 | 199651 | 179685.9 | 219616.1 | 161717.31 | 235588.18 | 334540 | 301086 | 367994 | 391411.8 | 277668.2 | 209876 | 188888.4 | 235061.12 | 157407 | 256048.72 |
| N | (20:2/18:0) Phosphatidylcholine | 0 | 0 | 0 | 0 | 0 | 2.00E+07 | 17983477.6 | 21979805.96 | 23378520.88 | 16584762.68 | 2627270.42 | 2364543.378 | 2942542.87 | 1970452.815 | 3205269.912 |
| N | 297.3 | 213467 | 192120.3 | 234813.7 | 172908.27 | 251891.06 | 223412 | 201070.8 | 245753.2 | 261392.04 | 185431.96 | 0 | 0 | 0 | 0 | 0 |
| N | 575.5 | 208765 | 187888.5 | 229641.5 | 169099.65 | 246342.7 | 0 | 0 | 0 | 0 | 0 | 0 | 0 | 0 | 0 | 0 |
| P | 5-hydroxyeicosatetraenoic | 5513700.345 | 4962330.311 | 6065070.38 | 4466097.279 | 6506166.407 | 1.67E+07 | 15072449.61 | 18421882.86 | 19594184.49 | 13900147.98 | 6309362.24 | 5678426.016 | 7066485.709 | 4732021.68 | 7697421.933 |
| P | 480 | 365412 | 328870.8 | 401953.2 | 295983.72 | 431186.16 | 309876 | 278888.4 | 340863.6 | 362554.92 | 257197.08 | 387654 | 348888.6 | 434172.48 | 290740.5 | 472937.88 |
| P | 592.2 | 1324509 | 1192058.1 | 1456959.9 | 1072852.29 | 1562920.62 | 1232408 | 1109167.2 | 1355648.8 | 1441917.36 | 1022898.64 | 0 | 0 | 0 | 0 | 0 |
| N | 504 | 213421 | 192078.9 | 234763.1 | 172871.01 | 251836.78 | 231241 | 208116.9 | 254365.1 | 270551.97 | 191930.03 | 209876 | 188888.4 | 235061.12 | 157407 | 256048.72 |
| P | 510.2 | 1471131.06 | 1324017.954 | 1618244.166 | 1191616.159 | 1735934.651 | 1920311.665 | 1728280.498 | 2112342.831 | 2246764.648 | 1593858.682 | 0 | 0 | 0 | 0 | 0 |
| P | 9-oxo-Octadecadienoic acid | 0 | 0 | 0 | 0 | 0 | 1878765 | 1690888.5 | 2066641.5 | 2198155.05 | 1559374.95 | 0 | 0 | 0 | 0 | 0 |
| N | 508 | 287654 | 258888.6 | 316419.4 | 232999.74 | 339431.72 | 223124 | 200811.6 | 245436.4 | 261055.08 | 185192.92 | 187659 | 168893.1 | 210178.08 | 140744.25 | 228943.98 |
| N | 512.2 | 409872 | 368884.8 | 450859.2 | 331996.32 | 483648.96 | 342310 | 308079 | 376541 | 400502.7 | 284117.3 | 145470 | 130923 | 162926.4 | 109102.5 | 177473.4 |
| N | 514.2 | 345120 | 310608 | 379632 | 279547.2 | 407241.6 | 223413 | 201071.7 | 245754.3 | 261393.21 | 185432.79 | 167650 | 150885 | 187768 | 125737.5 | 204533 |
| N | 526.2 | 354678 | 319210.2 | 390145.8 | 287289.18 | 418520.04 | 134560 | 121104 | 148016 | 157435.2 | 111684.8 | 169808 | 152827.2 | 190184.96 | 127356 | 207165.76 |
| N | 528.2 | 113456 | 102110.4 | 124801.6 | 91899.36 | 133878.08 | 132456 | 119210.4 | 145701.6 | 154973.52 | 109938.48 | 76765 | 69088.5 | 85976.8 | 57573.75 | 93653.3 |
| N | 336.2 | 954343 | 858908.7 | 1049777.3 | 773017.83 | 1126124.74 | 454343 | 408908.7 | 499777.3 | 531581.31 | 377104.69 | 821231 | 739107.9 | 919778.72 | 615923.25 | 1001901.82 |
| N | 353.1 | 945231 | 850707.9 | 1039754.1 | 765637.11 | 1115372.58 | 545231 | 490707.9 | 599754.1 | 637920.27 | 452541.73 | 454310 | 408879 | 508827.2 | 340732.5 | 554258.2 |
| N | 380.2 | 76532 | 68878.8 | 84185.2 | 61990.92 | 90307.76 | 0 | 0 | 0 | 0 | 0 | 0 | 0 | 0 | 0 | 0 |
| N | 339.3 | 132456 | 119210.4 | 145701.6 | 107289.36 | 156298.08 | 146789 | 132110.1 | 161467.9 | 171743.13 | 121834.87 | 126789 | 114110.1 | 142003.68 | 95091.75 | 154682.58 |
| N | 1083 | 567866 | 511079.4 | 624652.6 | 459971.46 | 670081.88 | 589990 | 530991 | 648989 | 690288.3 | 489691.7 | 285667 | 257100.3 | 319947.04 | 214250.25 | 348513.74 |
| N | 1087 | 287654 | 258888.6 | 316419.4 | 232999.74 | 339431.72 | 276551 | 248895.9 | 304206.1 | 323564.67 | 229537.33 | 187653 | 168887.7 | 210171.36 | 140739.75 | 228936.66 |
| N | 1091 | 497610 | 447849 | 547371 | 403064.1 | 587179.8 | 765412 | 688870.8 | 841953.2 | 895532.04 | 635291.96 | 344441 | 309996.9 | 385773.92 | 258330.75 | 420218.02 |
| P | 1047 | 198765 | 178888.5 | 218641.5 | 160999.65 | 234542.7 | 209876 | 188888.4 | 230863.6 | 245554.92 | 174197.08 | 66543 | 59888.7 | 74528.16 | 49907.25 | 81182.46 |
| P | 924.3 | 256431 | 230787.9 | 282074.1 | 207709.11 | 302588.58 | 273412 | 246070.8 | 300753.2 | 319892.04 | 226931.96 | 267812 | 241030.8 | 299949.44 | 200859 | 326730.64 |
| P | 926.4 | 654323 | 588890.7 | 719755.3 | 530001.63 | 772101.14 | 453213 | 407891.7 | 498534.3 | 530259.21 | 376166.79 | 631245 | 568120.5 | 706994.4 | 473433.75 | 770118.9 |
| P | 947.34 | 267512 | 240760.8 | 294263.2 | 216684.72 | 315664.16 | 232123 | 208910.7 | 255335.3 | 271583.91 | 192662.09 | 234123 | 210710.7 | 262217.76 | 175592.25 | 285630.06 |
| P | 1091 | 123214 | 110892.6 | 135535.4 | 99803.34 | 145392.52 | 223123 | 200810.7 | 245435.3 | 261053.91 | 185192.09 | 123241 | 110916.9 | 138029.92 | 92430.75 | 150354.02 |
| P | 991.2 | 364551 | 328095.9 | 401006.1 | 295286.31 | 430170.18 | 305666 | 275099.4 | 336232.6 | 357629.22 | 253702.78 | 123444 | 111099.6 | 138257.28 | 92583 | 150601.68 |
| N | 1107 | 345455 | 310909.5 | 380000.5 | 279818.55 | 407636.9 | 325455 | 292909.5 | 358000.5 | 380782.35 | 270127.65 | 124876 | 112388.4 | 139861.12 | 93657 | 152348.72 |
| N | 1131 | 176543 | 158888.7 | 194197.3 | 142999.83 | 208320.74 | 267564 | 240807.6 | 294320.4 | 313049.88 | 222078.12 | 154578 | 139120.2 | 173127.36 | 115933.5 | 188585.16 |
| N | 1035 | 1342450 | 1208205 | 1476695 | 1087384.5 | 1584091 | 1156434 | 1040790.6 | 1272077.4 | 1353027.78 | 959840.22 | 786564 | 707907.6 | 880951.68 | 589923 | 959608.08 |
| P | 1063 | 156432 | 140788.8 | 172075.2 | 126709.92 | 184589.76 | 70876 | 63788.4 | 77963.6 | 82924.92 | 58827.08 | 136432 | 122788.8 | 152803.84 | 102324 | 166447.04 |
| N | 1161 | 156540 | 140886 | 172194 | 126797.4 | 184717.2 | 73565 | 66208.5 | 80921.5 | 86071.05 | 61058.95 | 0 | 0 | 0 | 0 | 0 |
| P | 1040 | 75464 | 67917.6 | 83010.4 | 61125.84 | 89047.52 | 167589 | 150830.1 | 184347.9 | 196079.13 | 139098.87 | 0 | 0 | 0 | 0 | 0 |
| N | 1083 | 123450 | 111105 | 135795 | 99994.5 | 145671 | 15641 | 14076.9 | 17205.1 | 18299.97 | 12982.03 | 14321 | 12888.9 | 16039.52 | 10740.75 | 17471.62 |
| N | 632.1 | 127653 | 114887.7 | 140418.3 | 103398.93 | 150630.54 | 576551 | 518895.9 | 634206.1 | 674564.67 | 478537.33 | 287653 | 258887.7 | 322171.36 | 215739.75 | 350936.66 |
| N | 854.2 | 304441 | 273996.9 | 334885.1 | 246597.21 | 359240.38 | 765412 | 688870.8 | 841953.2 | 895532.04 | 635291.96 | 97654 | 87888.6 | 109372.48 | 73240.5 | 119137.88 |
| P | 880.2 | 56543 | 50888.7 | 62197.3 | 45799.83 | 66720.74 | 209876 | 188888.4 | 230863.6 | 245554.92 | 174197.08 | 66543 | 59888.7 | 74528.16 | 49907.25 | 81182.46 |
| P | 788.2 | 601245 | 541120.5 | 661369.5 | 487008.45 | 709469.1 | 232123 | 208910.7 | 255335.3 | 271583.91 | 192662.09 | 234123 | 210710.7 | 262217.76 | 175592.25 | 285630.06 |

*Abbreviations: MG means model group, NG means control group, SG means *S. platensis* group.

**Data are expressed as the average of three determinations (n=5).

**Table S5: Metabolite pathway changes for differential metabolites involved in RA.**

| **Pathway Name** | **p** | **-log(p)** | **Holm p** | **FDR** | **Impact** |
| --- | --- | --- | --- | --- | --- |
| [Alanine, aspartate and glutamate metabolism](https://www.metaboanalyst.ca/MetaboAnalyst/Secure/pathway/PathResultView.xhtml) | 1.30E-05 | 4.8877 | 0.0010879 | 0.0010879 | 0.2476 |
| [Citrate cycle (TCA cycle)](https://www.metaboanalyst.ca/MetaboAnalyst/Secure/pathway/PathResultView.xhtml) | 6.92E-05 | 4.16 | 0.0057424 | 0.0029058 | 0.21148 |
| [Arginine biosynthesis](https://www.metaboanalyst.ca/MetaboAnalyst/Secure/pathway/PathResultView.xhtml) | 5.23E-04 | 3.2818 | 0.042858 | 0.012467 | 0.11675 |
| [Butanoate metabolism](https://www.metaboanalyst.ca/MetaboAnalyst/Secure/pathway/PathResultView.xhtml) | 6.48E-04 | 3.1882 | 0.052509 | 0.012467 | 0 |
| [Arachidonic acid metabolism](https://www.metaboanalyst.ca/MetaboAnalyst/Secure/pathway/PathResultView.xhtml) | 7.42E-04 | 3.1295 | 0.059369 | 0.012467 | 0.33373 |
| [D-Glutamine and D-glutamate metabolism](https://www.metaboanalyst.ca/MetaboAnalyst/Secure/pathway/PathResultView.xhtml) | 0.0020748 | 2.683 | 0.16391 | 0.029047 | 0.5 |
| Histidine metabolism | 0.017038 | 1.7869 | 1 | 0.20446 | 0.22131 |
| [Sphingolipid metabolism](https://www.metaboanalyst.ca/MetaboAnalyst/Secure/pathway/PathResultView.xhtml) | 0.02603 | 1.5845 | 1 | 0.31236 | 0.1785 |
| [Glutathione metabolism](https://www.metaboanalyst.ca/MetaboAnalyst/Secure/pathway/PathResultView.xhtml) | 0.044529 | 1.3514 | 1 | 0.46756 | 0.02675 |
| [Glyoxylate and dicarboxylate metabolism](https://www.metaboanalyst.ca/MetaboAnalyst/Secure/pathway/PathResultView.xhtml) | 0.056759 | 1.246 | 1 | 0.5001 | 0.03175 |
| [Linoleic acid metabolism](https://www.metaboanalyst.ca/MetaboAnalyst/Secure/pathway/PathResultView.xhtml) | 0.059881 | 1.2227 | 1 | 0.5001 | 0 |
| [Glycerophospholipid metabolism](https://www.metaboanalyst.ca/MetaboAnalyst/Secure/pathway/PathResultView.xhtml) | 0.050037 | 1.1947 | 1 | 0.5001 | 0.11182 |
| [Nitrogen metabolism](https://www.metaboanalyst.ca/MetaboAnalyst/Secure/pathway/PathResultView.xhtml) | 0.071443 | 1.146 | 1 | 0.5001 | 0 |
| [Fatty acid biosynthesis](https://www.metaboanalyst.ca/MetaboAnalyst/Secure/pathway/PathResultView.xhtml) | 0.11102 | 0.95461 | 1 | 0.69009 | 0 |
| [Aminoacyl-tRNA biosynthesis](https://www.metaboanalyst.ca/MetaboAnalyst/Secure/pathway/PathResultView.xhtml) | 0.11502 | 0.93924 | 1 | 0.69009 | 0 |
| [alpha-Linolenic acid metabolism](https://www.metaboanalyst.ca/MetaboAnalyst/Secure/pathway/PathResultView.xhtml) | 0.14868 | 0.82775 | 1 | 0.83261 | 0 |
| [Pyruvate metabolism](https://www.metaboanalyst.ca/MetaboAnalyst/Secure/pathway/PathResultView.xhtml) | 0.23907 | 0.62148 | 1 | 1 | 0 |
| [Pentose phosphate pathway](https://www.metaboanalyst.ca/MetaboAnalyst/Secure/pathway/PathResultView.xhtml) | 0.23907 | 0.62148 | 1 | 1 | 0.04712 |
| [Propanoate metabolism](https://www.metaboanalyst.ca/MetaboAnalyst/Secure/pathway/PathResultView.xhtml) | 0.24853 | 0.60462 | 1 | 1 | 0 |
| [Glycolysis / Gluconeogenesis](https://www.metaboanalyst.ca/MetaboAnalyst/Secure/pathway/PathResultView.xhtml) | 0.27625 | 0.5587 | 1 | 1 | 2.10E-04 |
| [Tryptophan metabolism](https://www.metaboanalyst.ca/MetaboAnalyst/Secure/pathway/PathResultView.xhtml) | 0.31171 | 0.50625 | 1 | 1 | 0 |
| [Cysteine and methionine metabolism](https://www.metaboanalyst.ca/MetaboAnalyst/Secure/pathway/PathResultView.xhtml) | 0.33721 | 0.47209 | 1 | 1 | 0.10446 |
| [Biosynthesis of unsaturated fatty acids](https://www.metaboanalyst.ca/MetaboAnalyst/Secure/pathway/PathResultView.xhtml) | 0.36182 | 0.4415 | 1 | 1 | 0 |
| [Arginine and proline metabolism](https://www.metaboanalyst.ca/MetaboAnalyst/Secure/pathway/PathResultView.xhtml) | 0.37775 | 0.4228 | 1 | 1 | 0.086 |
| [Tyrosine metabolism](https://www.metaboanalyst.ca/MetaboAnalyst/Secure/pathway/PathResultView.xhtml) | 0.40847 | 0.38884 | 1 | 1 | 0.02463 |
| [Steroid biosynthesis](https://www.metaboanalyst.ca/MetaboAnalyst/Secure/pathway/PathResultView.xhtml) | 0.40847 | 0.38884 | 1 | 1 | 0.0282 |
| [Primary bile acid biosynthesis](https://www.metaboanalyst.ca/MetaboAnalyst/Secure/pathway/PathResultView.xhtml) | 0.43775 | 0.35877 | 1 | 1 | 0.05065 |
| [Steroid hormone biosynthesis](https://www.metaboanalyst.ca/MetaboAnalyst/Secure/pathway/PathResultView.xhtml) | 0.65974 | 0.18063 | 1 | 1 | 0.00528 |

*Raw P represents the original P value calculated from the enrichment analysis. Holm P represents the P value further adjusted using Holm-Bonferroni method. FDR P represents the P value adjusted using false discovery rate. The magnitude of the enrichment factor indicates the reliability of significance.


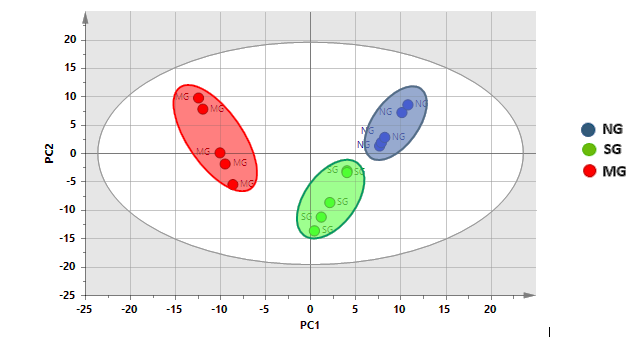


**Figure S3:** PCA score diagram based on serum metabolic profiles of normal group (NG), RA model group (MG) and *Spirulina* group (SG).

References

1. Kumar, N., Singh, S., Patro, N. & Patro, I. Evaluation of protective efficacy of Spirulina platensis against collagen-induced arthritis in rats. *Inflammopharmacology* **17**, 181–190 (2009).

2. Ali, E. A. I., Barakat, B. M. & Hassan, R. Antioxidant and angiostatic effect of Spirulina platensis suspension in complete Freund’s adjuvant-induced arthritis in rats. *PLoS One* **10**, e0121523 (2015).
